# *Congolius*, a new genus of African reed frog endemic to the central Congo: A potential case of convergent evolution

**DOI:** 10.1038/s41598-021-87495-2

**Published:** 2021-04-16

**Authors:** Tadeáš Nečas, Gabriel Badjedjea, Michal Vopálenský, Václav Gvoždík

**Affiliations:** 1 Czech Academy of Sciences, Institute of Vertebrate Biology, Květná 8, 603 65 Brno, Czech Republic; 2 Department of Botany and Zoology, Faculty of Science, Masaryk University, Kotlářská 2, 611 37 Brno, Czech Republic; 3 Biodiversity Monitoring Centre, Department of Ecology and Biodiversity of Aquatic Resources, University of Kisangani, Avenue Munyororo 550, Kisangani, Democratic Republic of the Congo; 4 Czech Academy of Sciences, Institute of Theoretical and Applied Mechanics, Prosecká 76, 190 00 Prague, Czech Republic; 5 Department of Zoology, National Museum, Cirkusová 1740, 193 00 Prague, Czech Republic

**Keywords:** Herpetology, Phylogenetics, Speciation, Taxonomy

## Abstract

The reed frog genus *Hyperolius* (Afrobatrachia, Hyperoliidae) is a speciose genus containing over 140 species of mostly small to medium-sized frogs distributed in sub-Saharan Africa. Its high level of colour polymorphism, together with in anurans relatively rare sexual dichromatism, make systematic studies more difficult. As a result, the knowledge of the diversity and taxonomy of this genus is still limited. *Hyperolius robustus* known only from a handful of localities in rain forests of the central Congo Basin is one of the least known species. Here, we have used molecular methods for the first time to study the phylogenetic position of this taxon, accompanied by an analysis of phenotype based on external (morphometric) and internal (osteological) morphological characters. Our phylogenetic results undoubtedly placed *H. robustus* out of *Hyperolius* into a common clade with sympatric *Cryptothylax* and West African *Morerella*. To prevent the uncovered paraphyly, we place *H. robustus* into a new genus, *Congolius*. The review of all available data suggests that the new genus is endemic to the central Congolian lowland rain forests. The analysis of phenotype underlined morphological similarity of the new genus to some *Hyperolius* species. This uniformity of body shape (including cranial shape) indicates that the two genera have either retained ancestral morphology or evolved through convergent evolution under similar ecological pressures in the African rain forests.

## Introduction

African reed frogs, Hyperoliidae Laurent, 1943, are presently encompassing almost 230 species in 17 genera. These are split into two subfamilies, Hyperoliinae Laurent, 1943 with 12 genera, and the less speciose subfamily Kassininae Laurent, 1972 with 5 genera^1,2^ (Table 1). The phylogenetic relationships among the hyperoliid frogs are still not completely understood, and the systematics has undergone recent changes, e.g., revalidation of the subfamily Kassininae and reclassification of *Acanthixalus* to this subfamily^1^ or genus-level reorganizations in some species^3^. There is also a number of genera whose classification to the family is clear on the basis of morphological characters, but their exact phylogenetic position is still an unanswered question. In the Congo Basin, an important region for our study, it is the case of three monotypic genera. *Callixalus pictus* Laurent, 1950 and *Chrysobatrachus cupreonitens* Laurent, 1951 were described from high-altitude bamboo forests and grasslands of the Albertine Rift^4,5^, and *Kassinula wittei* Laurent, 1940 is known from wooded savanna uplands of south-eastern Democratic Republic of the Congo, northern Zambia, and recently from central Angola^6,7^. Some previous morphological studies provided rough ideas where these genera might be phylogenetically placed^8,9^, but the first two genera have never been studied by a molecular approach. All three genera are presently placed in Hyperoliinae^1,2^, neverthless, the hypothesized sister relationship of *Callixalus* and *Acanthixalus*^8,9^ keeps the question on the phylogenetic position of *Callixalus* open. Similar uncertainties apply to *Chrysobatrachus* and *Kassinula*. However, the latter has a characteristically different morphology, resembling either *Kassina*^6,10^, *Paracassina*^8^, or *Afrixalus*^7,9^. Newly, *Kassinula* was shown to be phylogenetically related to *Afrixalus*, possibly even embed within this genus^7^. A recent surprise in the hyperoliid systematics was the discovery of *Morerella cyanophthalma* Rödel, Assemian, Kouamé, Tohé & Perret, 2009, a monotypic genus from the coastal rain forests of Côte d’Ivoire in West Africa^11^. The study included phylogenetic analysis of three mitochondrial markers, which did place the genus in Hyperoliidae, but did not resolve its exact systematic position. Later studies, which comprised phylogenetic or phylogenomic analyses of nuclear DNA, uncovered affiliation of *Morerella* to *Cryptothylax* from Central African rain forests^1,3^.

**Table 1:** Genera of the family Hyperoliidae, including their species richness and distribution^2^.

Subfamily	Genus	Spp	Distribution
Hyperoliinae Laurent, 1943	*Afrixalus* Laurent, 1944	35	sub-Saharan Africa
	*Alexteroon* Perret, 1988	3	western Central Africa
	*Arlequinus* Perret, 1988	1	western Central Africa
	*Callixalus* Laurent, 1950	1	Albertine Rift
	*Chrysobatrachus* Laurent, 1951	1	Albertine Rift
	*Cryptothylax* Laurent & Combaz, 1950	2	Central Africa
	*Heterixalus* Laurent, 1944	11	Madagascar
	*Hyperolius* Rapp, 1842	145	sub-Saharan Africa
	*Kassinula* Laurent, 1940	1	southern Central Africa
	*Morerella* Rödel, Kosuch, Grafe, Boistel & Veith, 2009	1	West Africa
	*Opisthothylax* Perret, 1966	1	western Central Africa
	*Tachycnemis* Fitzinger, 1843	1	Seychelles Islands
Kassininae Laurent, 1972	*Acanthixalus* Laurent, 1944	2	West and Central Africa
	*Kassina* Girard, 1853	15	sub-Saharan Africa
	*Paracassina* Peracca, 1907	2	North-eastern Africa
	*Phlyctimantis* Laurent & Combaz, 1950	5	West, Central and East Africa
	*Semnodactylus* Hoffman, 1939	1	southern Africa

**Figure 1:**
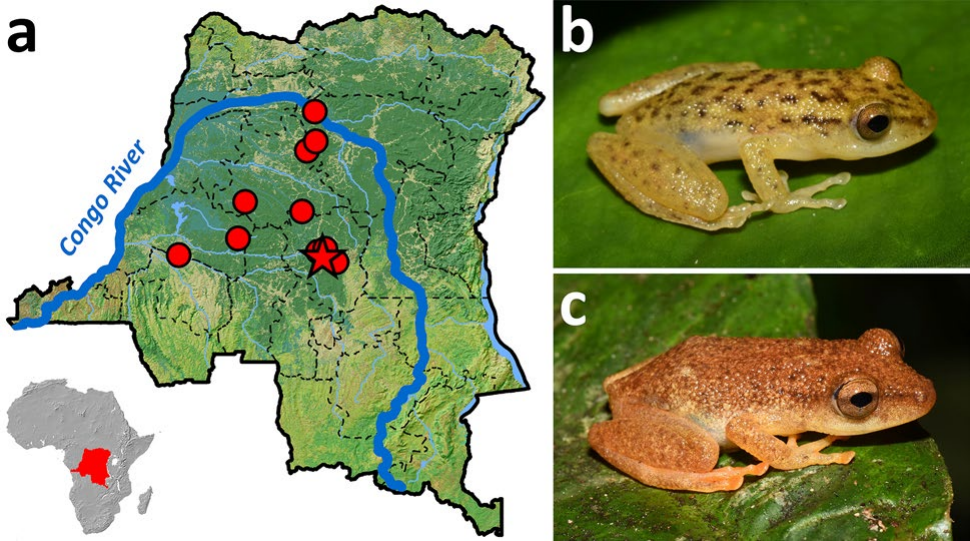
*Hyperolius robustus*—now in the new genus *Congolius*—and its distribution. (**a**) All known localities lie to the south of the wide arc of the Congo River (left bank), within the Democratic Republic of the Congo; star denotes type locality. Photographs of (**b**) adult male (IVB-H-CD18-143) and (**c**) adult female (NMP-P6V 76087/2) from the Kokolopori Bonobo Nature Reserve. Photos by V.G.

More than a half of all hyperoliids belong to the genus *Hyperolius* distributed throughout sub-Saharan Africa^2, 12^. *Hyperolius* is a genus of mostly medium-sized and exclusively arboreal frogs with a great variation in colouration between and within species, often sexually dichromatic (otherwise rare in anurans), and with remarkably conservative morphology^13^. It is a genus inhabiting wide range of habitats from open savannas and bushland to closed-canopy rain forests^13^. One of the rain forest dwelling species is the little-known *Hyperolius robustus* Laurent, 1979 (Fig. 1 and Supplementary Fig. S1). It was described from a handful of localities in the central Congo Basin, Sankuru region of the present-day Democratic Republic of the Congo (DRC)^14^. Since the description, this species has only been reported by one author^13,15^, and only scarce biological data exist.

*Hyperolius robustus* is one of the larger species in the genus, with both sexes reaching up to 37–38 mm in body length^13,15^. Dark yellow to brown dorsum with varying intensity of dark spots in males differs from a reddish-brown dorsal colouration in females (Fig. 1b,c and Supplementary S2). Males bear large flat gular gland (disc, flap) of yellowish to white colour. The venter is coloured yellow in males and orange in females, especially limbs. *Hyperolius robustus* can be distinguished from its sympatric congeners by proportions of the head, especially relatively longer snout and closely-positioned nostrils. It resembles members of the *H. concolor* species group, specifically *H. balfouri* (Werner, 1908), *H. cinnamomeoventris* Bocage, 1866, or *H. kivuensis* Ahl, 1931 when compared to the species widespread across the Congo^14^. While Laurent^14^ suggested its probable relationship to the *H. concolor* group, he also noted that some of its morphological characters supported a possible close relationship to *Cryptothylax*, a genus superficially also resembling the *H. concolor* group, although reaching a larger adult size^14^. A later publication focusing more broadly on external morphology of *Hyperolius* gained an additional support for the similarity of *H. robustus* to *H. balfouri* and *H. cinnamomeoventris*^16^.

Recently, we collected new specimens of *H. robustus* at multiple sites in the central Congolian forests, and reviewed available museum material, including types (Supplementary Table S1, Appendices S1 and S2). This study aims to (1) clarify the phylogenetic and systematic position of *Hyperolius robustus* using a molecular multilocus approach, and (2) evaluate morphological variation based on morphometric and osteological approaches in a comparative framework.

## Results

### Molecular analyses

#### Conventional phylogenetics

**Figure 2:**
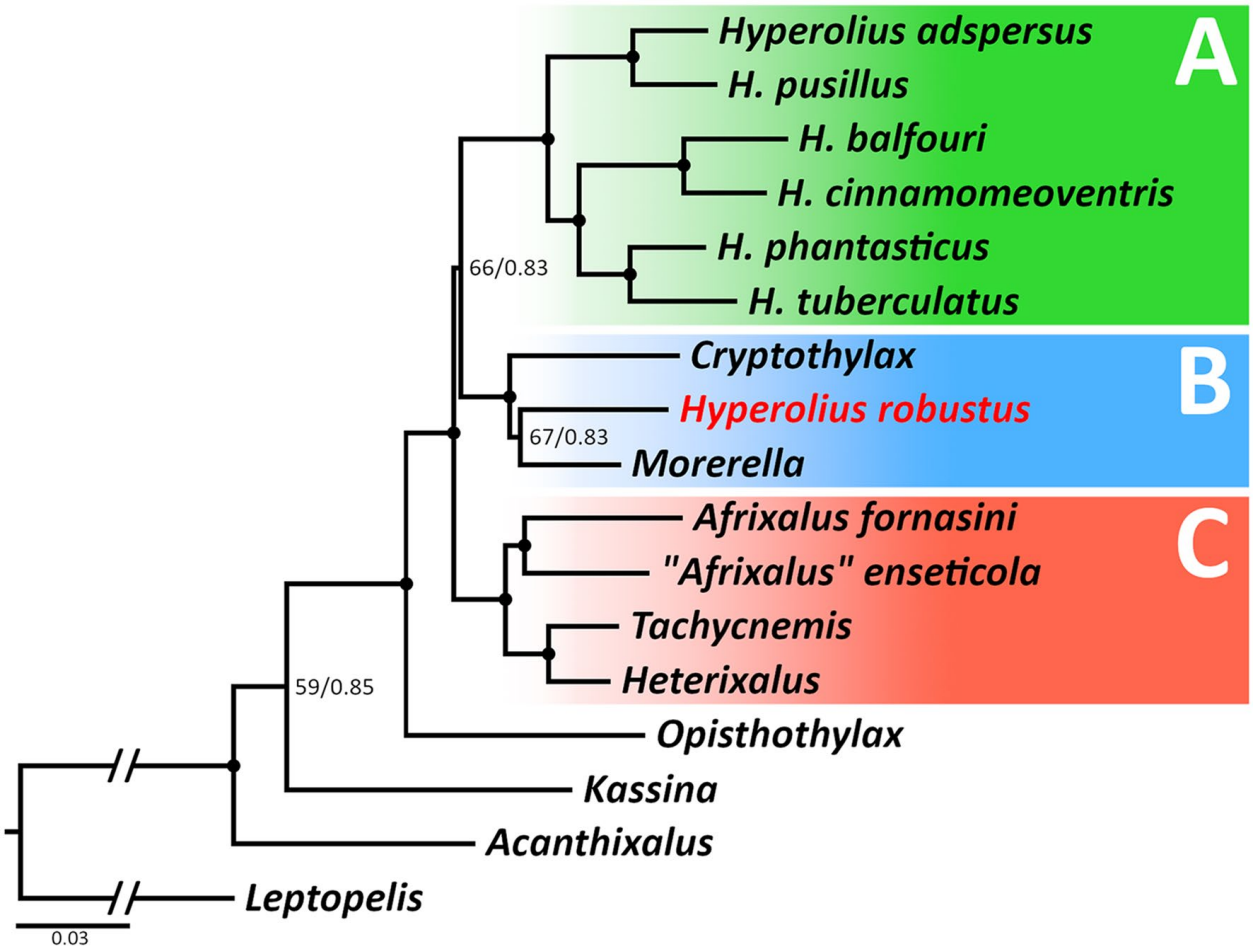
Phylogenetic tree of hyperoliid frogs with a focus on the subfamily Hyperoliinae. The majority rule consensus tree inferred from Bayesian analyses of four nuclear and one mitochondrial marker (complete data set). Dots at nodes represent high supports (ML bootstrap ≥ 70/Bayesian posterior probability ≥ 0.95) while numbers show support values for nodes with a lower support. Sequence GenBank accession numbers are provided in Supplementary Table S1.

The phylogenetic analysis of the complete concatenated data set of four nuclear and one mitochondrial marker places *H. robustus* well outside the clade containing the genus *Hyperolius* (Fig. 2), rendering *Hyperolius* paraphyletic. The tree based on this complete concatenated data set has only minor difference in the topology compared to the tree based on the nuclear-only data set (Supplementary Fig. S3f). However, support values are lower in the complete data set. All phylogenetic inferences of individual markers or concatenated data put the taxa from the subfamily Hyperoliinae into a common clade, with variable support values depending on a molecular marker/data set. General topology within Hyperoliinae is also consistent across the markers/data sets (except of the mitochondrial tree, which has a lower resolution; Supplementary Fig. S3a). *Opisthothylax* is positioned as a basal genus in the subfamily Hyperoliinae and in the sister position to all other available Hyperoliinae genera forming three main clades (hereafter Clades A–C). *Hyperolius robustus* together with *Cryptothylax* and *Morerella* form the well-supported Clade B (received the full support in the complete and nuclear-only concatenated data sets), which is in the sister position to Clade A containing the remaining *Hyperolius*. The relationships within Clade B are uncertain but the sister relationship between *H. robustus* and *Morerella* received a full support in the nuclear-only data set. Clade A (*Hyperolius* sensu stricto) is a well-supported clade composed of three sub-clades. One subclade contains *H. adspersus* and *H. pusillus* (= *H. nasutus* complex^15^), and is in the sister position to the remaining two subclades. Another subclade (Clade 2 sensu Portik et al*.*^1^) is represented by *H. balfouri* and *H.* cf. *cinnamomeoventris* (an unresolved species complex of multiple distinct lineages^17^; hereafter as *H. cinnamomeoventris*) and is in the well-supported sister position to the third clade (Clade 1 sensu Portik et al*.*^1^), which includes *H. phantasticus* and *H. tuberculatus* (a similar case as the *H. cinnamomeoventris* species complex^17^; hereafter as *H. tuberculatus*). The third main clade, the well-supported Clade C contains *Afrixalus* from sub-Saharan Africa, *Heterixalus* from Madagascar, and *Tachycnemis* from Seychelles. Support values for the relationships within the subfamily Hyperoliinae (ingroup), among the three main Clades A–C, differ depending on a particular ML and BI analysis (Fig. 2 and Supplementary Fig. S3). However, all these analyses provide a strong support for the position of *H. robustus* outside of the genus *Hyperolius.* All nuclear markers, including the concatenated data set, place *H. robustus* into a common clade with *Cryptothylax* and *Morerella*, with three of the four nuclear markers (and the nuclear concatenated data set) supporting the sister relationship of *H. robustus* and *Morerella* with an intermediate to high support (only one marker infers the sister relationship between *Cryptothylax* and *Morerella* with an intermediate support). The phylogenetic analysis of the relatively short *16S* mitochondrial marker places *H. robustus* in the sister position to *Opisthothylax*, but with a low support.

#### Dated species tree

**Figure 3:**
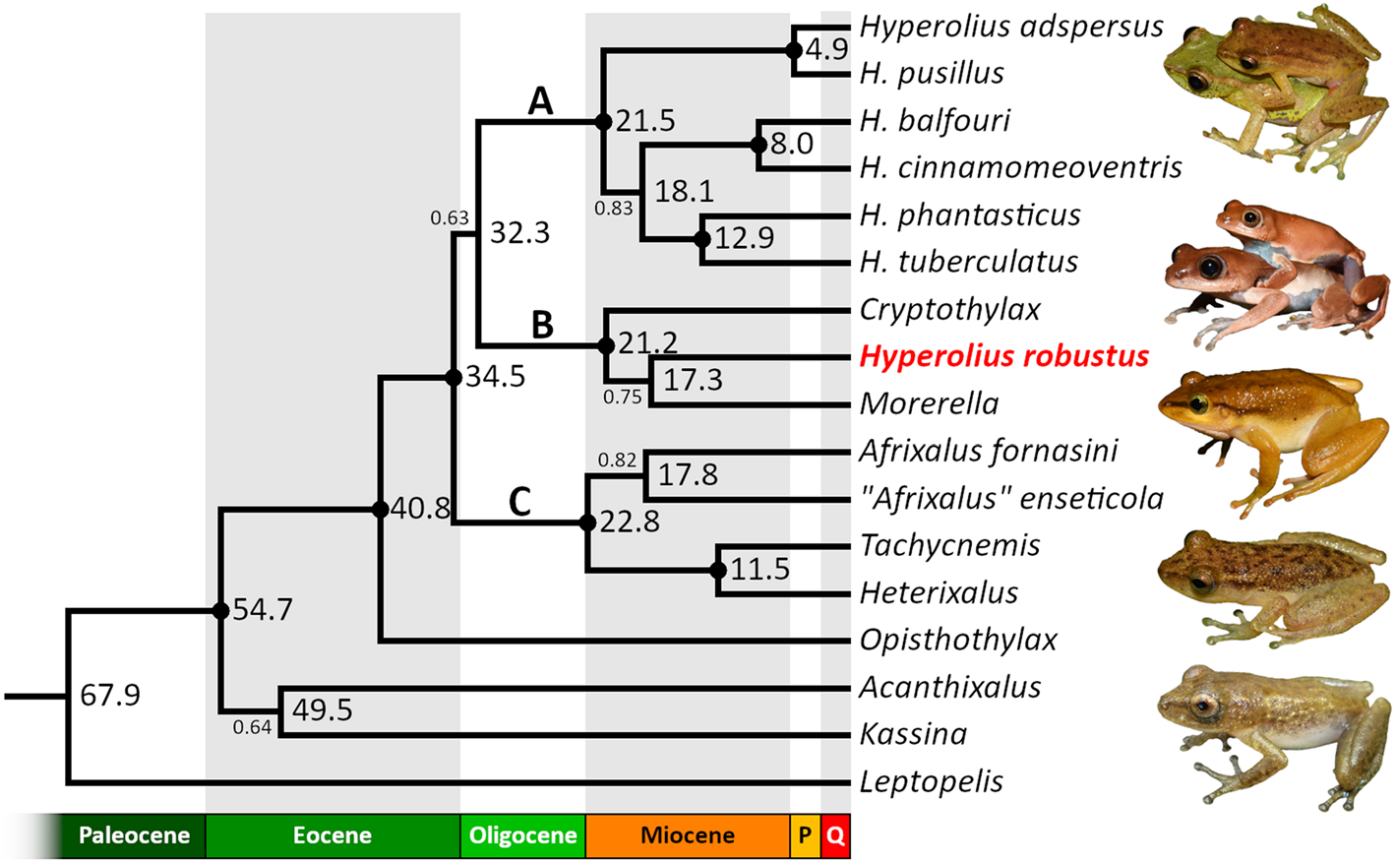
Time-calibrated species tree of the subfamily Hyperoliinae. Maximum clade credibility tree based on StarBEAST2 analysis of four nuclear markers with geological epochs as a time scale (abbreviation P stands for Pliocene and Q for Quaternary). Dots at nodes signify high supports (Bayesian posterior probability ≥ 0.95), median node ages are shown to the right from each node, lower support values are shown to the left of the relevant node. *Leptopelis* (Arthroleptidae) is outgroup. Photos on the right side from top to bottom (not to scale): *Hyperolius cinnamomeoventris*, *H. phantasticus*, *Cryptothylax greshoffii* (in a calling position; note the extensive gular gland and missing vocal sac), *H. robustus* (now *Congolius robustus* gen. et comb. n.), *Morerella cyanophthalma*. Photos by V.G. and T. F. Kpan (*Morerella cyanophthalma*).

The species-tree analysis yielded a tree with a similar topology as the conventional phylogenetic analyses (Fig. 3). *Opisthothylax* is placed with a high support in a basal position within the Hyperoliinae subfamily and its lineage diverged at approx. 40.8 Mya (± 5.5) in the middle Eocene. Similarly to the conventional phylogenetic analyses, all three main Clades A–C are well supported but their mutual relationships are not resolved. The diversification into the three main clades occurred during the Eocene–Oligocene transition (approx. 28–39 Mya, mean 33.4 Mya). Clade B received the full support, and—as in a majority of the conventional phylogenetic inferences—*H. robustus* is placed in the sister relationship to *Morerella*, albeit without a high support. The diversification within Clade B is dated in the early Miocene (21.2 ± 4.9 Mya), with the split between *H. robustus* and *Morerella* inferred at around 17.3 Mya (± 7.2). Clade A (the remaining *Hyperolius*) is also well supported, forming three subclades, but the sister relationship between the *balfouri–cinnamomeoventris* and *phantasticus–tuberculatus* subclades lacks a higher support (contrary to the conventional analyses). The initial radiation of the remaining studied *Hyperolius* began at around 21.5 Mya (± 3.7) in the early Miocene. The well-supported Clade C containing *Afrixalus*, *Heterixalus*, and *Tachycnemis* diversified approx. 22.8 Mya (± 5.1) in the early Miocene. The sister relationship of the two included *Afrixalus* species received only a weaker support, with the divergence dated to 17.8 Mya (± 8.6) in the Miocene. In fact, one of the *Afrixalus* species (*A. enseticola*) probably represents a distinct genus^1^. The youngest divergence between valid genera of Hyperoliinae was found between *Heterixalus* and *Tachycnemis* dated to11.5 Mya (± 5.8) in the late to middle Miocene.

### Systematic account

Hereafter, we describe a new genus for *H. robustus* to remove the uncovered paraphyly of *Hyperolius*. To justify its genus level distinction, beside its specific morphological characters (see below), we refer to the deeper phylogenetic divergence than is found between *Heterixalus* from Madagascar and *Tachycnemis* from Seychelles, and a comparable divergence between *Afrixalus* (*A. fornasini* is type species of the genus) and a candidate, yet undescribed genus containing “*Afrixalus*” *enseticola*^1^.

Family Hyperoliidae Laurent, 1943.

Subfamily Hyperoliinae Laurent, 1943.

***Congolius *** genus novum Nečas, Badjedjea & Gvoždík.

ZooBank registration: urn:lsid:zoobank.org:act:7C7D57F2-3D02-4456–9292-184A98A559E6.

Type species: *Hyperolius robustus* Laurent, 1979.

Suggested English name: Congo Frog.

Content: *Congolius robustus* (Laurent, 1979) **combinatio nova** (formerly *Hyperolius robustus*), Robust Congo Frog. Presently a monotypic genus.

#### Etymology

The generic name *Congolius* is of the masculine gender and it is a compound of the name Congo, referring to the region where the genus is endemic to, and the Greek word *eleios* = smooth in the Latinized form as the suffix –*lius*, which is used in the genus name *Hyperolius* to refer to its former affiliation of *Congolius robustus* to this genus.

#### Diagnosis and comparisons

**Figure 4:**
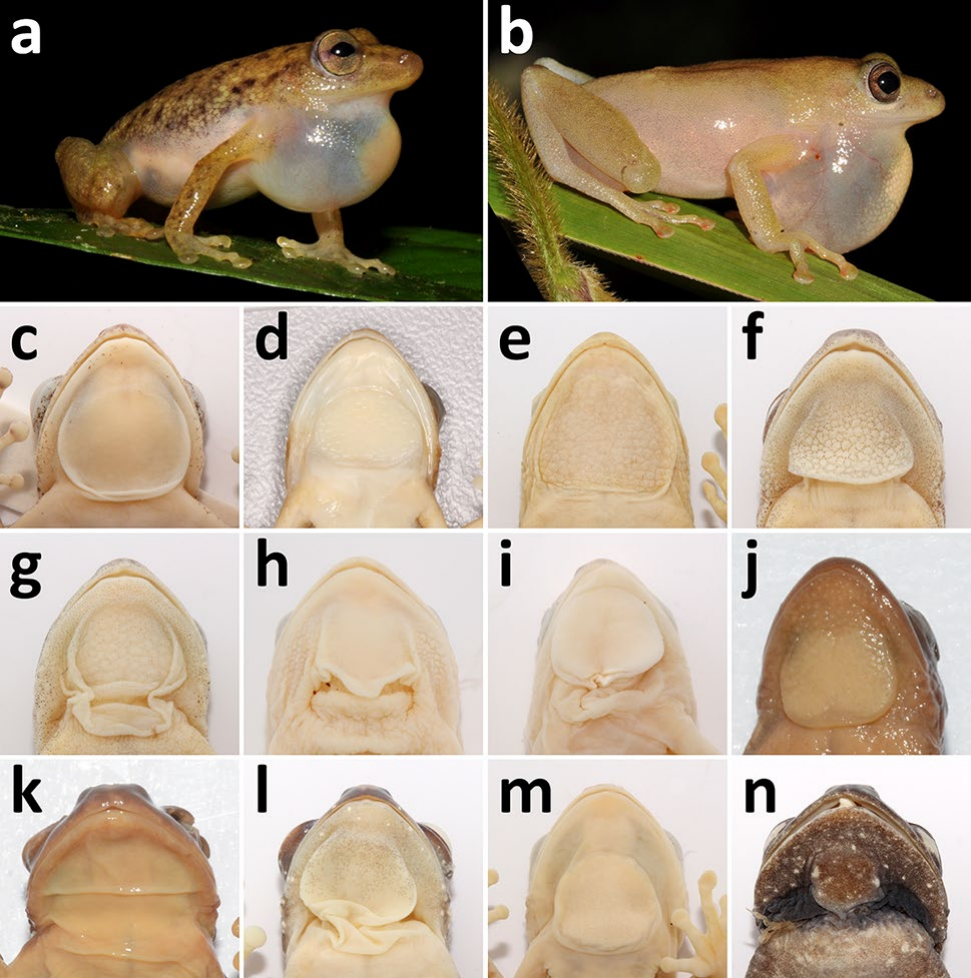
Gular region of selected hyperoliid taxa. (**a**) *Congolius robustus* gen. et comb. n. (IVB-H-CD18-255) with inflated vocal sac, (**b**) *Hyperolius balfouri* (IVB-H-CD15-062) with inflated vocal sac, (**c**) *C. robustus* gen. et comb. n. (ZMUC-R.771175), (**d**) *Morerella cyanophthalma* (ZMB 71588, paratype), (**e**) *Cryptothylax greshoffii* (NMP-P6V 76076/1), (**f**) *H. balfouri* (IVB-H-CD15-061), (**g**) *H.* cf. *cinnamomeoventris* (IVB-H-CD18-092), (**h**) *H. phantasticus* (IVB-H-CD18-203), (**i**) *H.* cf. *adspersus* (IVB-H-CD15-139), (**j**) *Chrysobatrachus cupreonitens* (MCZ A-64739, paratype), (k) *Callixalus pictus* (MCZ A-64707), (**l**) *Afrixalus equatorialis* (NMP-P6V 76073/1), (**m**) *Opisthothylax immaculatus* (NMP-P6V 76090/1), (**n**) *Phlyctimantis verrucosus* (NMP-P6V 76085/1). Photos by (**a**,**b**,**j**,**k**) V.G., (**c**,**e**–**i**,**l**–**n**) T.N. and (**d**) F. Tillack and M.-O. Rödel.

The new genus *Congolius* can be distinguished from other genera within the family Hyperoliidae by following characters: pupil horizontal (vertical or rhomboidal in *Acanthixalus*, *Afrixalus*, *Arlequinus*, *Cryptothylax*, *Heterixalus*, *Kassina*, *Kassinula*, *Tachycnemis*, *Opisthothylax*, *Paracassina*, *Phlyctimantis*, and *Semnodactylus*; predominately horizontal but sometimes rhomboidal in *Morerella*, see photos in^18^); tympanum indistinct but visible in both sexes (indistinct in *Acanthixalus*, *Callixalus*, *Chrysobatrachus*, *Heterixalus*, *Kassinula*, *Opisthothylax*, and in most *Hyperolius* and *Afrixalus*); males with large gular gland with free lateral and posterior margins without free skin folds of dilatable skin beneath, Fig. 4c [gular gland present in males of all hyperoliid species; paired, laterally positioned in *Acanthixalus*; large without free margins obscuring whole gular region in *Cryptothylax* (Fig. 4e); positioned anteromedially and hidden under semi-transparent integument in *Alexteroon*; transverse in *Arlequinus*; rather indistinct transverse oval composed of two elements fused medially in *Callixalus* (Fig. 4k); distinct round composed of two elements fused medially in *Chrysobatrachus* (Fig. 4j); in a form of round median disc without extended dilatable skin beneath or around present in *Morerella* (Fig. 4d), *Opisthothylax* (Fig. 4m) and *Tachycnemis*; medioposteriorly positioned with free skin folds beneath the posterior margin in *Afrixalus* (Fig. 4l), *Heterixalus*, and most *Hyperolius* (Fig. 4f–i; exceptions exist in *Hyperolius*, e.g. *H. zonatus* Laurent, 1958 from West Africa;^12^); longitudinal oval or strap-shaped with free lateral margins in *Kassina*; round with free lateral margins in *Paracassina*, *Phlyctimantis* (Fig. 4n), and *Semnodactylus*]; dilatable vocal sac (pouch) present, when inflated forms a hemisphere extending to the pectoral region, Fig. 4a (extensively dilatable vocal sac absent in *Arlequinus*, *Acanthixalus*, *Callixalus*, *Chrysobatrachus*, *Cryptothylax*, and *Morerella*; see Fig. 4b for comparison with inflated vocal sac of *Hyperolius balfouri*); limbs without skin “fringes” (present in *Alexteroon*); dorsal skin slightly granular in both sexes (tuberculous in *Acanthixalus*); sexually dichromatic (colouration difference between sexes similar to *Cryptothylax* and *Morerella*, where females display more orange to reddish colouration than males, see Fig. 1b,c and Supplementary Fig. S2; sexually monochromatic genera: *Acanthixalus*, *Afrixalus*, *Alexteroon*, *Arlequinus*, *Callixalus*, *Chrysobatrachus*, some *Heterixalus*, some *Hyperolius*, *Kassina*, *Kassinula*, *Opisthothylax*, *Paracassina*, *Phlyctimantis*, and *Semnodactylus*).

Osteologically, *Congolius* is characterized with sphenethmoid not dorsally exposed (dorsally visible in *Cryptothylax*, some *Hyperolius*, *Kassina*, *Kassinula*, *Phlyctimantis*, *Semnodactylus*, and *Tachycnemis*); ventroanterior portion of sphenethmoid unfused (fused in some *Kassina*, *Paracassina*, some *Phlyctimantis*, and *Semnodactylus*); praemaxilla and maxilla bear minute teeth; overlapping of praemaxilla and maxilla is minimal; nasals triangular, separated medially from each other and posteriorly from frontoparietals; canthal regions of nasals rounded; vomers lack teeth-bearing dentigerous processes, “vomerine teeth” (present in *Cryptothylax*, most *Kassina*, and *Phlyctimantis*); frontoparietals rectangular; quadratojugal anteriorly in contact with maxilla (not in contact with maxilla in *Acanthixalus*, some *Afrixalus*, *Callixalus*, *Chrysobatrachus*, some *Heterixalus*, some *Hyperolius*, some *Kassina*, and *Kassinula*; reduced or absent in some *Afrixalus*); columella present (reduced in *Callixalus*); neural arches non-imbricate (imbricate in *Cryptothylax*, *Kassina*, *Paracassina*, *Phlyctimantis*, and *Semnodactylus*); transverse processes of 8^th^ vertebra angled perpendicularly to vertebral column (angled markedly forward in some *Afrixalus*, some *Hyperolius*, some *Kassina*, *Kassinula*, *Opisthothylax*, and *Semnodactylus*); relative length of vertebral column (ratio of length of vertebral column to transverse processes of 8th presacral vertebra) between values of 1.6 and 2.4 (longer vertebral column in *Afrixalus*, *Callixalus*, some *Heterixalus*, some *Hyperolius*, *Kassina*, *Kassinula*, *Paracassina*, *Phlyctimantis*, and *Semnodactylus*); medial margins of coracoids entire (centrally perforated in *Kassinula*, and *Paracassina*); omosternum greatly forked (unforked or less forked in *Callixalus*, some *Hyperolius*, and *Paracassina*); base of metasternum ossified (completely ossified in *Morerella*); carpal and tarsal bones not fused (Hyperoliidae synapomorphy); subarticular sesamoids absent (present in *Kassina*, *Paracassina*, some *Phlyctimantis*, and *Semnodactylus*); intercalary elements completely mineralized (cartilaginous centres in *Afrixalus*, *Callixalus*, *Heterixalus*, and *Hyperolius*); terminal phalanges long and peniform (long and claw-shaped in some *Heterixalus*, some *Hyperolius*, some *Kassina*, *Kassinula*, and *Opisthothylax*; short obtuse in some *Kassina*, *Paracassina*, and *Phlyctimantis*; bifurcated in *Acanthixalus*, some *Kassina*, and possibly *Arlequinus* based on external morphology, no osteological data for the latter). Osteological terminology follows Drewes^8^. See Supplementary Appendix S3 for literature resources.

Genetically, *Congolius* is differentiated, forms a clade with *Cryptothylax* and *Morerella*, and differs from other genera in the family Hyperoliidae by an uncorrected *p*-distance larger than 11.0% in the fragment of 16S rRNA (Supplementary Table S2).

#### Distribution

*Congolius robustus* is probably endemic to the central Congolian lowland forests, to the south of the wide arc of the Congo River (Fig. 1a). It has never been recorded from the right bank of the Congo River. For locality details, see Supplementary Appendix S2. It occupies occasionally flooded forests and dense farmbush along small streams. *Congolius robustus* has a nocturnal activity, and it can typically be found perching on vegetation 1.5–2 m high.

#### Nomenclatural note

This publication and the nomenclatural act are registered in ZooBank, the online Official Register of Zoological Nomenclature. The LSID (Life Science Identifier) of this publication from ZooBank is urn:lsid:zoobank.org:pub:F2045C29-D703-4FC8-B317-1BFD09815E15 and can be accessed at https://zoobank.org/.

### Morphology

#### External morphology

**Figure 5:**
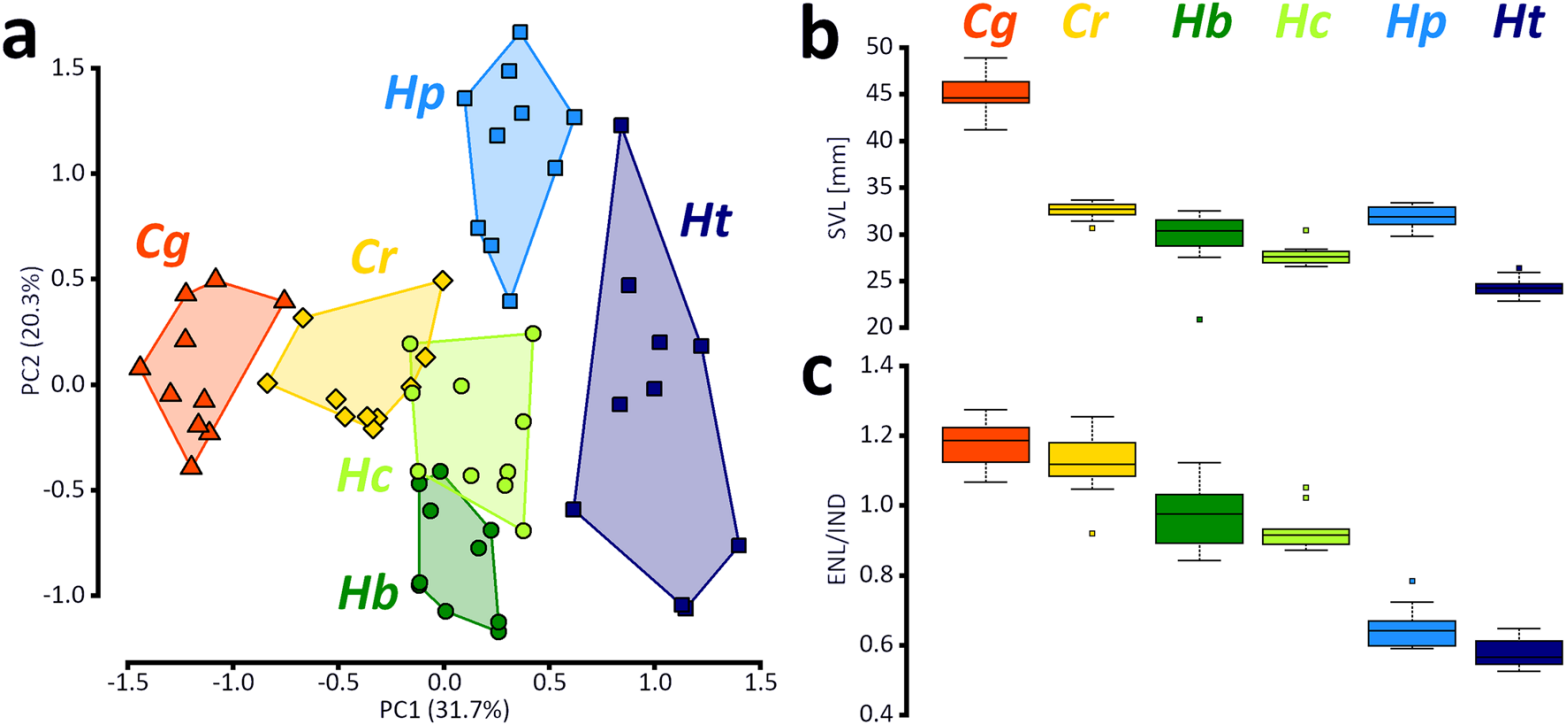
Morphometric analyses of external characters of *Congolius* gen. n. and relative (*Cryptothylax*) or similar (*Hyperolius*) genera from the lowland Congo Basin. Four *Hyperolius* species represent two phylogenetic clades (see Methods for details). (**a**) Principal component analysis of body shape, (**b**) variation of SVL as boxplots, (**c**) variation of the ENL/IND ratio as boxplots. Boxplots display average values (line), upper and lower quartiles (box), minimums and maximums without outliers (whiskers), and outliers (dots). *Cg* (*Cryptothylax greshoffii*), *Cr* (*Congolius robustus* gen. et comb. n.), *Hb* (*Hyperolius balfouri*), *Hc* (*H. cinnamomeoventris*), *Hp* (*H. phantasticus*), and *Ht* (*H. tuberculatus*).

Body size of the six investigated taxa as average snout-vent length (SVL ± SD; in mm) in adult males is: *Congolius robustus* (32.5 ± 0.9), *Cryptothylax greshoffii* (45.2 ± 2.1), *Hyperolius balfouri* (29.4 ± 3.2), *H. cinnamomeoventris* (27.8 ± 1.0), *H. phantasticus* (32.0 ± 1.1), *H. tuberculatus* (24.4 ± 1.0) (Fig. 5b). Principal component analysis (PCA) was performed to evaluate body shape variation of the above-mentioned taxa. Factor loadings for the first three principal components (PCs) and most contributing variables are provided in Supplementary Table S3. The body shapes of the six taxa were found significantly different using MANOVA (F_5,15_ = 57.07, *p* =  < 2.2e−16). The PC1 (31.7% of explained variation) can be interpreted as showing mostly the shape of head, especially snout. The PC2 (20.3%) was influenced mostly by the head width and variables characterizing the length of limbs. The PCA (Fig. 5a) placed *Congolius* along the PC1 axis between *Cryptothylax* and the four *Hyperolius* species in close proximity to *H. cinnamomeoventris*. The PC2 does not differentiate *Congolius* and *Cryptothylax* from representatives of *Hyperolius*, but differences among the species of the latter genus are apparent. The studied taxa are well separated from each other in the morphospace (PC1 x PC2), except of small overlaps of *H. cinnamomeoventris* with *Congolius* and *H. balfouri* putting the three taxa into a common cluster. The main distinguishing morphometric characters between *Congolius* and *Hyperolius* are the characters related to the shape of snout: eye-nostril distance (measured along body axis) is usually larger than internarial distance in *Congolius* (as well as *Cryptothylax*), and on average the same or smaller in the studied *Hyperolius*. The snout shape of the representatives of Clade 2 (*H. balfouri*, *H. cinnamomeoventris*) is more similar to *Congolius* than the snout shape of the representatives of Clade 1 (*H. phantasticus*, *H. tuberculatus*) (clades sensu Portik et al*.*^1^; Fig. 5c).

Larval morphology is not presently known.

#### Osteology

**Figure 6:**
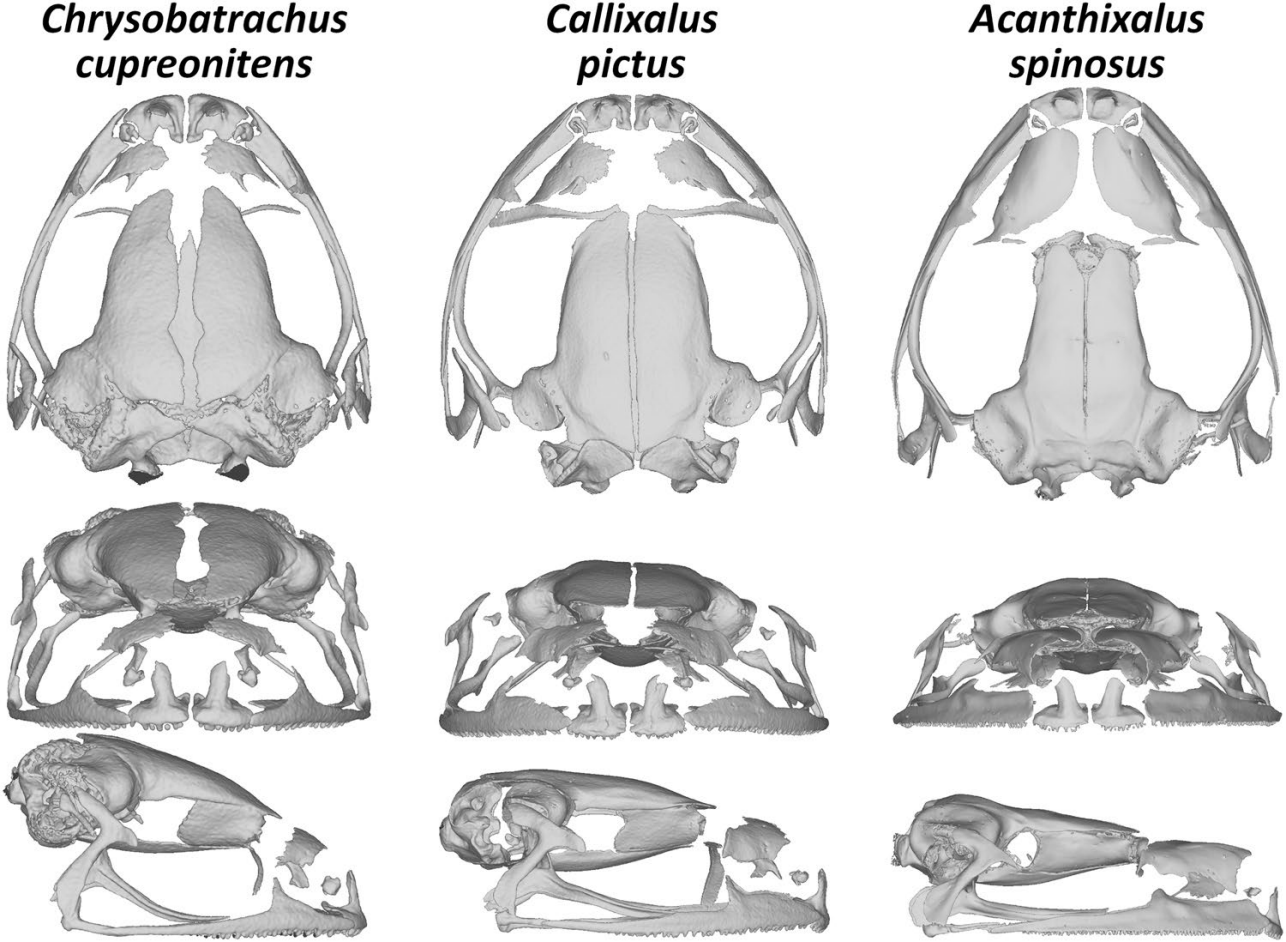
Cranial morphology of *Chrysobatrachus*, *Callixalus*, and *Acanthixalus*. Reconstructions in dorsal, frontal, and lateral views are based on high-resolution X-ray microcomputed tomography. From left to right: *Chrysobatrachus cupreonitens* (CAS 145263, male), *Callixalus pictus* (CAS 145260, male), and *Acanthixalus spinosus* (CAS 153800, female). Skull views are scaled to a similar size among the three genera.

**Figure 7:**
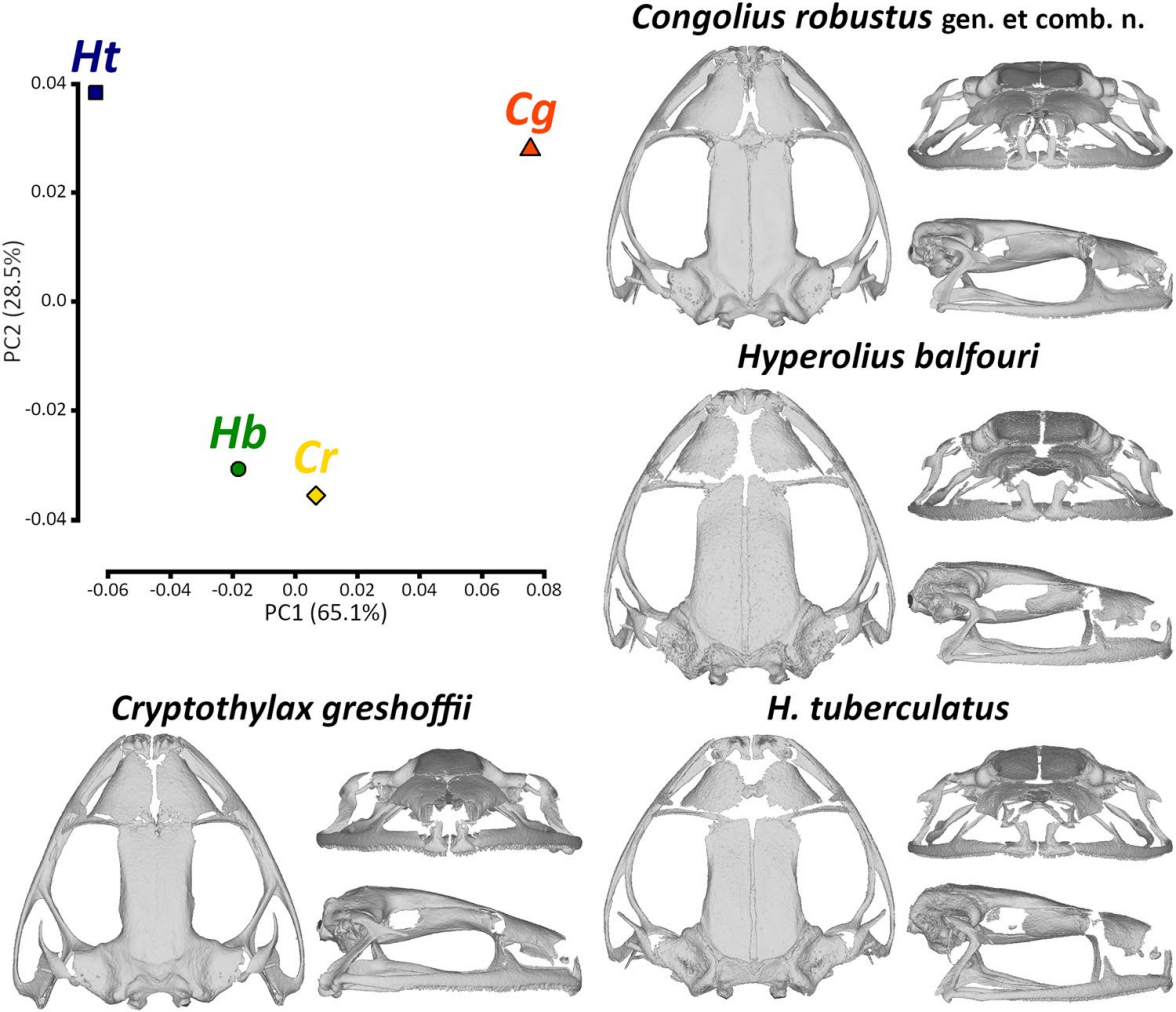
Cranial morphology and shapes in a morphospace of *Congolius* gen. n. and relative (*Cryptothylax*) or similar (*Hyperolius*) genera from the lowland Congo Basin. The morphospace is expressed by a principal component analysis of geometric morphometric landmarks. *Cg* (*Cryptothylax greshoffii*, IVB-H-CD15-169, male), *Cr* (*Congolius robustus* gen. et comb. n., ZMUC-R.771175, male), *Hb* (*Hyperolius balfouri*, IVB-H-CD15-061, male), and *Ht* (*H. tuberculatus*, IVB-H-CD15-010, male). Reconstructions in dorsal, frontal, and lateral views are based on high-resolution X-ray microcomputed tomography. Skull views are scaled to a similar size among the four taxa. See Supplementary Fig. S4 for comparison of all scanned specimens.

Skeletons with a special focus on skulls were studied in *Congolius*, its relative *Cryptothylax*, *Hyperolius*, two little-known genera *Chrysobatrachus* and *Callixalus*, and *Acanthixalus*. All are distributed in the Congo Basin, and thus of a systematic importance in respect to our erection of the new genus. Scans of skulls are shown in Figs. 6 and 7, for a female of *C. robustus* see Supplementary Figs. S4, S6. No differences were found in the osteological characters between male and female *C. robustus*. Our new osteological data were used in addition to the previously published^8^ for genus-level comparisons (see “Diagnosis and comparisons”). A special focus on the little-known genera distributed in the Congo uncovered that the skeletons of *Acanthixalus,* *Chrysobatrachus* and *Callixalus* exhibited number of characters clearly differentiating them from the new genus *Congolius*: bifurcated phalanges, and flat frontoparietals in *Acanthixalus*; moderately forked omosternum in *Callixalus*; reduced nasals in *Callixalus* and *Chrysobatrachus*; cartilaginous metasternum, and short and tall skull in *Chrysobatrachus* [8] (this study).

Cranial data of *Congolius* and the remaining examined taxa, relative (*Cryptothylax*) or similar (*Hyperolius*) genera, were examined using the 3D geometric morphometrics. PCA generated two relevant PCs (Fig. 7), while the third contributed only 0.06% of the explained variation. PCA scores of the four analysed taxa are in Supplementary Table S4. The PC1 (65.1%) axis could be explained as a change of the cranial shape from rounded (negative PC1 values) to more triangular (positive PC1 values) from the dorsoventral view, including a shift of the mandibular joint to more posterior position or an elongation of frontoparietals and nasals (Fig. 7). *Congolius* lays in a close proximity to *H. balfouri*, while *H. tuberculatus* and *Cryptothylax* are positioned more distantly in different directions. The PC2 (28.5%) axis explains a change in the shape from a rather prolonged skull (negative PC2 values) to a wider skull (positive PC2 values). The examined taxa are clustered into two groups along the PC2 axis, *Congolius* and *H. balfouri* (negative PC2 scores), and *Cryptothylax* and *H. tuberculatus* (positive PC2 scores).

## Discussion

The phylogenetic analyses confirmed our initial finding that “*Hyperolius robustus*”, now *Congolius robustus*, is phylogenetically placed outside of *Hyperolius*, as well as Laurent’s^14^ hypothesis of the possible relationship to *Cryptothylax greshoffii*. These two Central African taxa and *Morerella cyanophthalma* from West Africa form a common clade (Clade B), which is in the sister relationship to *Hyperolius* (Clade A, Figs. 2, 3). According to our results, the West African monotypic genus *Morerella* is the closest relative to *Congolius* diverging around 17 Mya (Figs. 2, 3). However, the sister relationship of the two genera is not always highly supported. For example, the analysis of the all-markers data set shows only a medium support for this relationship (Fig. 2). However, this could be caused by the implementation of the *16S* mitochondrial marker, which is not optimal for deeper phylogenies due to saturated substitutions (see also Supplementary Fig. S3a). The phylogenetic analysis of the nuclear-markers data set gives to the *Congolius–Morerella* clade a high support (Supplementary Fig. S3f). A close relationship of *Congolius* and *Morerella* is further suggested by their similar superficial morphology as well as osteological characters. In particular, these two genera share a similar expression of sexual dichromatism (males brownish, females reddish), the general morphology of the gular region (lack of visible extended dilatable skin beneath or around gular gland), and the general osteological composition (the only main difference is in the degree of metasternum ossification).

The general topology of our phylogenetic reconstruction of the subfamily Hyperoliinae corresponds to the results of a recent phylogenomic study^1^. The only exception is “*Afrixalus*” *enseticola*, which is not in our results placed in the same clade with the genera *Heterixalus* and *Tachycnemis*^1^, but shares a clade with *A. fornasini* (type species of the genus). However, the divergence is relatively deep and the support only intermediate (Figs. 2, 3, Supplementary Fig. S3f.). The diversification into the three major clades in the subfamily Hyperoliinae (Clades A–C) took place during the Eocene–Oligocene transition (around 33 Mya). The Eocene–Oligocene boundary is associated with an extinction event on Earth^19–21^. This event is attributed to a rapid decrease in the global temperature caused mainly by the opening of the southern oceanic gateway, Earth orbital cycles, and a decrease of the atmospheric CO_2_ levels^22–26^. Most notably on the African continent, it was the accompanying aridification^23,27^. Our analyses lack a higher support in resolving the relationships among the three main clades, which suggests consecutive divergences in a short period of time. Such extinctions as at the Eocene–Oligocene transition might have opened previously occupied niches and could have led to radiations in vacant places in the ecosystem. The radiation within the clade B, which contains *Congolius*, is estimated to occur at around 21–17 Mya (Fig. 3). Similar ages were estimated for the radiations of Clades A and C occurring in the early Miocene (approx. 23–18 Mya). The Miocene is a geological epoch connected to yet another event of rapid global cooling at the Oligocene–Miocene transition (23 Mya)^23,28^. Although periods of the late Oligocene and the first half of Miocene were relatively warm (26 to 15 Mya)^23^, the mid-Miocene’s closure of the Tethys Ocean and continual northward drift of the African continent had major aridification effects^23,28^. Both events led to the reduction of the rainforest cover to refugia along coasts and major rivers by the late Miocene^27^. The rainforest withdrawal and fragmentation could have been a driver for the divergence in a common ancestor of Central African *Congolius* and West African *Morerella* occurring at approximately 17 Mya.

Representatives of the little-known genera *Callixalus* and *Chrysobatrachus* were not available for the molecular analyses. However, they were morphologically examined to compare with *Congolius*, including osteology (Fig. 6). Both genera, together with *Acanthixalus* from Kassininae, were found to have distinct morphological characters from the newly established genus (e.g., maxilla not in contact with quadratojugal). A possible close relationship of *Acanthixalus* and *Callixalus* was suggested earlier^8,9^ based on thorough morphological analyses. While a recent phylogenomic study placed *Acanthixalus* in Kassininae^1^, the monotypic genus *Callixalus* has conservatively remained in Hyperoliinae^1,2^, awaiting a focused study. *Callixalus* has not been recorded since its description in 1950 and hence, no recent genetic material is available. Our examination of *Callixalus* and *Acanthixalus* supports their similarity in a number of shared characters. However, we refrain from making systematic conclusions for these two genera, as our study was not designed to solve this question. A future research on the phylogenetic position of *Callixalus*, as well as *Chrysobatrachus* (rediscovered in 2011^29^), *Alexteroon* (revision of the genus is in preparation^30^), *Arlequinus*, and *Kassinula* is needed to reveal a complete genus-level phylogenetic reconstruction of Hyperoliidae.

The distribution range of *C. robustus* seems to be confined to the central Congolian forests, south of the wide arc of the Congo River. Several recent surveys did not report any sightings of *C. robustus* on the right bank of the Congo River^31–35^. Therefore, the Congo River may represent a barrier that prevents *C. robustus* from reaching the northern parts of the Congo Basin. In contrast, the Congo River does not seem to be a dispersal barrier for some *Hyperolius* species^17^. The barrier for *Congolius* to the east could be either the Lualaba River (= upper part of the Congo River) or the Lomami River which flows in parallel to the west. Future herpetological field research in the Lomami National Park, which lies between these two rivers, could bring an answer to this question.

Morphologically, *C. robustus* is highly resembling some *Hyperolius* species, in particular from the *H. concolor* group^14^. The morphometric analyses of external characters and the body shape showed a great similarity between phylogenetically rather distant taxa (Fig. 5). The main distinguishing morphological character is the shape of snout, being longer than wide in *Cryptothylax* and *Congolius* compared to *Hyperolius*. Although some specimens of *H. balfouri* and *H. cinnamomeoventris* reach similar values of the ENL/IND ratio (Fig. 5c). Our results generally support the findings of Laurent^14,16^, who already pointed out on the similarity of *Congolius* and these two species from the *H. concolor* group. The analysis of the shape of skull yielded very similar results. It put *Congolius* more closely to phylogenetically more distant *H. balfouri* than to a close relative, *Cryptothylax greshoffii*.

There are two evolutionary processes that could be responsible for the origin of the morphological similarity between the newly erected *Congolius* and some *Hyperolius* species. First is the morphological stasis, which postulates tendency of some lineages to retain ancestral ecological niches, and thus, to reduce the morphological change during an evolutionary lineage’s existence^36,37^. This hypothesis would suggests that *Congolius* and some lineages of *Hyperolius* may have retained the same general body shape as possessed by their common ancestor, i.e. since the beginning of the Oligocene (approx. 32 Mya; our dating corresponds to a previously published study^1^). Second is the convergent evolution, which evolves when the same or similar selection pressures cause independent development of analogous characters in unrelated taxa^38–40^. Convergent evolution is a common characteristic feature in species-rich communities, like are typically present in rain forests. An independent parallel evolution of a similar body shape in *Congolius* and some *Hyperolius* could have been driven by similar ecological pressures across the African rain forests. However, further investigations into causalities of ecological factors and the phenotypic evolution of African reed frogs are needed to understand processes leading to the morphological uniformity of some unrelated hyperoliid taxa.

## Conclusions

We present an evidence of the phylogenetic relationship of central Congolian “*Hyperolius*” *robustus* and sympatric *Cryptothylax*, as well as West African *Morerella*. We erect the new genus *Congolius* to prevent the uncovered paraphyly of *Hyperolius*, and place “*H.*” *robustus* to the new genus. *Congolius* morphologically greatly resembles some members of the genus *Hyperolius*, which might be attributable to either the morphological stasis or convergent evolution caused by similar ecological pressures in the African rain forests.

## Material and methods

### Sampling and molecular data

One museum^15^ and two recently collected “*Hyperolius” robustus* specimens from two locations (Kokolopori: ZMUC R.771176, IVB-H-CD18-255; Mombongo: NMP-P6V 76089; for details see Supplementary Table S1) were investigated genetically. Identifications were based on the comparison with the type material (Supplementary Fig. S1), the ZMUC material was already identified^15^. Preliminary phylogenetic analyses suggested that “*H.” robustus* might be placed out of *Hyperolius*. Taxon sampling was designed to focus on “*H.” robustus* and the subfamily Hyperoliinae (nucleotide sequences retrieved from GenBank; Supplementary Table S5), to include molecular data of all available genera (7/12), and representatives of main clades of *Hyperolius* and *Afrixalus* (sensu Portik et al*.*^1^). Two representatives of the hyperoliid subfamily Kassininae (*Acanthixalus spinosus*, *Kassina senegalensis*) and a representative of the sister family Arthroleptidae (*Leptopelis notatus*) were used as outgroups (retrieved from GenBank). Collection of osteological data focused on all systematically potentially important taxa from the Congo Basin, including those without molecular data (*Callixalus*, *Chrysobatrachus*). External morphometric examinations focused on Central African relative or morphologically similar taxa and selected “congeners” (*Hyperolius*) of “*H.” robustus*, see below for more details. Newly collected specimens were euthanized, fixed in 96% ethanol for a short period of time, and then transferred to 75% ethanol for preservation. Tissue samples for genetic analyses were extracted and fixed in 96% ethanol. Museum abbreviations follow the herpetological standards^41^ except for the abbreviation IVB (Institute of Vertebrate Biology of the Czech Academy of Sciences, Brno, Czech Republic) which is an institution registered with the Global Biodiversity Information Facility (http://grbio.org/cool/iskk-kcgq). IVB-H stands for a herpetological collection localised in the research facility Studenec.

DNA was extracted using a commercial DNA extraction kit following the manufacturer’s protocol (GeneAll Biotechnology). Single and two-step polymerase chain reactions (PCRs) were applied to amplify a mitochondrial fragment encoding 16S rRNA (hereinafter as *16S*; 530 bp after trimming) and four nuclear markers: FIC Domain-containing gene (*FICD*; 529 bp), KIAA2013 gene (*KIAA2013*; 540 bp), Proopiomelanocortin gene (*POMC*; 595 bp) and Tyrosinase gene (*Tyr*; 532 bp). See Supplementary Table S6 for primers. PCR protocols followed original publications^42–46^ with the exception of 16S rRNA, where minor changes were applied^47^. The newly acquired sequences of all three “*H.” robustus* (now *Congolius robustus*) specimens were deposited in the online database GenBank (https://ncbi.nlm.nih.gov/genbank/). Sequences were checked by eye and assembled using Geneious Prime R11.0.3^48^. MAFFT v7.450 plug-in for Geneious Prime was used to align all sequences^49,50^. See Supplementary Table S5 for GenBank accession numbers.

### Phylogenetic inference and divergence dating

The three analysed samples of “*H. robustus*” from two distant locations exhibited only minor variations (*16S*: C/T on the 29^th^ and A/G on the 183^rd^ position, *FICD*: G/R on the 146^th^ position, *POMC*: T/Y on the 293^rd^ and C/M on the 313^th^ positions, *Tyr*: A/G on the 91^st^ position). To streamline interpretations of results, only one sample (IVB-H-CD18-255) was included in the final data sets. Maximum likelihood (ML) analyses and Bayesian inference (BI) were performed for each gene separately, concatenated nuclear markers, and concatenated all markers. ML analyses were performed in RAxML-NG v0.9.0^51^ and BI using MrBayes v3.2.6^52^, with substitution models and partition schemes selected in PartitionFinder 2 (following the Bayesian information criterion, BIC)^53^ (Supplementary Table S7). Nodal support values in ML analyses were received by the bootstrap method with the automatic bootstopping cut-off value 0.03, stopping at 200 replicates in all runs. All MrBayes analyses were run twice with four Markov chains Monte Carlo for 30 million generations with sampling every 3,000^th^ generation. First 25% of produced trees were discarded as a “burn-in”. Clades supported with ML bootstrap values ≥ 70 and BI posterior probability values ≥ 0.95 were considered highly supported^54^, while bootstrap values ≥ 60 and posterior probability values ≥ 0.80 as intermediate support.

A dated coalescent-based species tree was inferred in StarBEAST2 v2.6.2^55^ based on the four nuclear markers (*FICD*, *KIAA2013*, *POMC*, *Tyr*). A lognormal relaxed molecular clock and a normal distribution were used^56^. Calibration dates were taken as averages of recently published large phylogenomic studies of anuran amphibians, where calibrations were based on paleontological and geological records^1, 57–59^. Two calibration points were set: splits between Arthroleptidae and Hyperoliidae at ~ 64.5 (56.8–72.5) Mya, and Hyperoliinae and Kassininae at ~ 44.3 (36.8–51.8) Mya. The Yule tree model was set, and substitution models and partition schemes as suggested by PartitionFinder 2 (BIC) were applied (Supplementary Table S7). The analysis was run thrice for 100 million generations with sampling every 5,000^th^ generation. Tracer 1.7.1^60^ was used to inspect stationarity and ESS values for all parameters. The first 10% of samples from each analysis were discarded as “burn-in”, and the total of 54,000 remaining trees from all three analyses were combined in LogCombiner 1.7, and TreeAnnotator 2.6 (both available as part of the BEAST package^61^ at http://beast.bio.ed.ac.uk) was used to obtain a final species tree as a maximum clade credibility tree.

### External morphology

Variation in external morphology, with a special interest to evaluate the similarity of the newly discovered genus and *Hyperolius*, was investigated on a total of 60 adult male specimens of six taxa (10 from each): *Congolius robustus*, *Cryptothylax greshoffii*, and four *Hyperolius* species. As the latter is a speciose genus, we included representatives of two main *Hyperolius* clades^1^. Two taxa were selected as representatives of Clade 2^1^, the same taxa as identified earlier as the most similar to *C. robustus*^14,16^: *H. balfouri* and *H. cinnamomeoventris* from the *H. concolor* species group. Two other taxa were selected as representatives of Clade 1^1^: *H. tuberculatus* from the *H. viridiflavus* group^60^ and *H. phantasticus* from the *H. pardalis* group^62^). *Hyperolius cinnamomeoventris* and *H. phantasticus* were also selected for their sympatric occurrence with *C. robustus* in the Kokolopori Bonobo Nature Reserve, Tshuapa, DRC. Representatives of the *H. nasutus* group, the third main *Hyperolius* clade^1^, were omitted for their characteristically different morphology (elongated slender body, sharp nose, translucent green colouration, sexes of same size and colouration)^13,63^. *Cryptothylax greshoffii* was selected as a sympatric relative of *Congolius*, and a representative of the sister clade of *Hyperolius* [1, 3] (this study). A list of all examined specimens is available in Supplementary Table S1.

Snout-vent length (SVL) and the following 15 measurements were taken with a digital calliper XTline P13430 150 mm to the nearest 0.1 mm (Supplementary Fig. S5): snout-urostyle length (SUL), from snout tip to posterior edge of urostyle; head width (HW), at greatest head width in close proximity to posterior edge of jaw; head length (HDL), from snout tip to posterior edge of jaw, measured along body axis; interorbital distance (IOD), shortest distance between upper eyelids; eye diameter (ED), between anterior and posterior corners of eye; eye-nostril length (ENL), from anterior corner of eye to centre of nostril, measured along body axis; internarial distance (IND), between centres of nostrils; snout length (SL), from anterior corner of eye to snout tip, measured along body axis; humerus length (HL), from body wall to outer edge of elbow; radioulna length (RL), from elbow to proximal edge of most proximal palmar tubercle; hand length (HaL), from most proximal palmar tubercle to tip of fourth manual digit; femur length (FL), from centre of vent to outer edge of knee; tibiofibula length (TL), from outer edge of knee to outer edge of tibiotarsal articulation; foot length (FoL), from most proximal edge of inner metatarsal tubercle to tip of fourth pedal digit; disc width (DW), at greatest width of adhesive disc of fourth pedal digit. These measurements follow recent taxonomic publications on *Hyperolius*^64^, and publications on general anuran morphometry^65^.

The measured values were subsequently statistically treated according to Mosimann^66^ to filter out the effect of body size, see also Gvoždík et al.^67^ or Dolinay et al.^68^. Principal component analysis (PCA) was performed in the programming language R v3.6.3^69^ using R-package “vegan” v2.5–6^70^ to explore the morphospace of the body shapes. Multivariate analysis of variance (MANOVA) was performed on the first three principal components to test body shape differentiation among the taxa, and descriptive statistics of body size and selected morphometric indices was calculated via boxplots^69^.

### Osteology

A high-resolution X-ray microcomputed tomography (μCT) was used to compare osteological characters of *Congolius robustus* to other genera of Hyperoliinae present in the Congo Basin: *Hyperolius* (*H. balfouri*, *H.* cf. *tuberculatus*), *Cryptothylax*, *Chrysobatrachus*, and *Callixalus* (in order of initially presumed relationships or similarity^8,9,14,16^). *Afrixalus* and *Kassinula* were omitted for their clearly different morphology^8,9^. The μCT scan of *Acanthixalus* (Kassininae) was downloaded from MorphoSource (http://morphosource.org/Detail/MediaDetail/Show/media_id/13843) to allow a comparison to *Callixalus*, a little-known Congolian hyperoliid possibly related to this genus^8,9^. The X-ray computed tomography of the six Hyperoliinae taxa was conducted at the Institute of Theoretical and Applied Mechanics, Czech Academy of Sciences, Centre Telč, Czech Republic. A patented device TORATOM (European patent 2835631) was used together with an in-house developed software ToraBaC (developed in the Centre Telč, Institute of Theoretical and Applied Mechanics, Czech Academy of Sciences, Czech Republic; http://www.itam.cas.cz/Software/Torabac/index.html) for a flat field correction, defective pixel corrections, and a projection equalization. Scans were conducted with a resolution of 20 μm/px at 120 kV voltage and 83 μA target current with rotation step of 0.15° (2400 projections per full rotation, no averaging) and exposure time of 280 ms. Volume Graphics VGStudio Max 3.2 (Volume Graphics GmbH, Heidelberg, Germany) was used for reconstruction and visualization. Examination and terminology of osteological characters followed Drewes^8^. All acquired scans were deposited in MorphoSource (https://morphosource.org/, see Data availability for details) in a form of BMP image stacks. See Supplementary Table S1 for details on the osteological material.

To examine, if the similarity of the new genus and *Hyperolius* has its basis in osteological characters, we evaluated a variation in the cranial shape of *Congolius* gen. n., and relative (*Cryptothylax*) or similar (*Hyperolius*) genera from the lowland Congo Basin. Thirty-two morphometric landmarks were selected following Paluh et al*.*^71^ (Supplementary Fig. S6). 3D landmark coordinates were digitized using Stratovan Checkpoint v2018.06.18 (Stratovan Corporation, Davis, U.S.A.), and Procrustes and principal component analyses were performed in R using package “geomorph” v3.2.1^72^ to evaluate the morphospace of the skull shape.

### Graphics

Map shown in the Fig. 1a was produced using ArcGIS v10.6 (Esri Inc. 2017) with implementation of GlobCover 2009^73^, and country boundaries downloaded from https://naturalearthdata.com. Images of skulls were produced in MeshLab v2020.05^74^. Photographs used were taken by authors if not stated otherwise. The final compositions and edits of all figures were done using GNU Image Manipulation Program v2.10.4 downloaded from https://gimp.org.

### Ethics statement

Examined museum specimens were loaned from museums with permissions from the curators of the herpetological collections. Collection and handling of live animals was approved by the institutional review board of the Biodiversity Monitoring Centre, University of Kisangani, Democratic Republic of the Congo. All experiments were carried out in accordance with relevant guidelines and regulations. This study meets all the conditions of the ARRIVE guidelines.

## Supplementary Information

Supplementary Information.

## Data Availability

The sequences obtained in this study are available in the GenBank online database (https://ncbi.nlm.nih.gov/genbank). IVB-H-CD18-255: MW626918, MW626921, MW626924, MW626927, MW626930; NMP-P6V 76089: MW626917, MW626920, MW626923, MW626926, MW626929; ZMUC R.771176: MW626916, MW626919, MW626922, MW626925, MW626928. The X-ray microcomputed tomography scans of the skulls obtained in this study are available in the MorphoSource online database (https://morphosource.org) in a form of BMP image stacks: *Callixalus pictus* CAS 145260: https://www.morphosource.org/biological_specimens/000S31537; *Chrysobatrachus cupreonitens* CAS 145263: https://www.morphosource.org/biological_specimens/000S31531; *Congolius robustus* ZMUC-R.771175 (male): https://www.morphosource.org/biological_specimens/000S31541; *Congolius robustus* ZMUC-R.771176 (female): https://www.morphosource.org/biological_specimens/000S31542; *Cryptothylax greshoffii* IVB-H-CD15-169: https://www.morphosource.org/biological_specimens/000S31540; *Hyperolius balfouri* IVB-H-CD15-061: https://www.morphosource.org/biological_specimens/000S31539; *Hyperolius* cf. *tuberculatus* IVB-H-CD15-010: https://www.morphosource.org/biological_specimens/000S31538. The μCT scan of *Acanthixalus spinosus* CAS 153800 was downloaded from https://www.morphosource.org/biological_specimens/000S10942, and used with the approval of the authors.
