# Supplementary material for: Congolius, a new genus of African reed frog endemic to the central Congo: A potential case of convergent evolution
Source: Sci Rep. 2021 Apr 16;11:8338. doi: 10.1038/s41598-021-87495-2 (PMC8052363; doi:10.1038/s41598-021-87495-2)
Supplement: Supplementary file 1 — Supplementary Information. [file 41598_2021_87495_MOESM1_ESM.pdf]

www.nature.com/scientificreports

SCIENTIFIC REPORTS
natureresearch

Congolius, a new genus of African reed frog endemic to the central Congo: A potential case of convergent evolution

Tadeáš Nečas*, Gabriel Badjedjea, Michal Vopálenský & Václav Gvoždík*

*Corresponding authors: T. N. (email: tad.necas@gmail.com) and V. G. (email: vaclav.gvozdik@gmail.com)

Supplementary material

Content **Page**

Fig. S1. Holotype and allotype of *Hyperolius robustus* Laurent, 1979. 1

Fig. S2. Sexual dichromatism in *Congolius robustus* gen. et comb. n. 2

Table S1. Main material examined. 3

Appendix S1. Additional material examined. 6

Appendix S2. Distribution records of *Congolius robustus* gen. et comb. n. 7

Fig. S3. Bayesian phylogenetic trees of (a–e) individual genetic markers, and (f) concatenated data set based on four nuclear markers. 8

Appendix S3. Resources for comparative diagnosis of *Congolius* gen. n. with other genera of the family Hyperoliidae. 9

Table S2. Uncorrected *p*-distances based on a fragment of the 16S rRNA gene. 10

Table S3. Factor loadings of the first three principal components of PCA of body shape. 11

Fig. S4. Cranial morphology of all osteologically examined taxa. 12

Table S4. Factor scores of the first two principal components of PCA of cranial shape. 14

Table S5. Taxa and samples included in phylogenetic analyses with GenBank accession numbers. 15

Table S6. Primers used to amplify selected molecular markers. 16

Table S7. Substitution models used in phylogenetic analyses. 17

Fig. S5. Visualization of 16 measured characters. 18

Fig. S6. Visualization of 32 digitized landmarks. 19

Appendix S4. References used in Supplementary material. 20

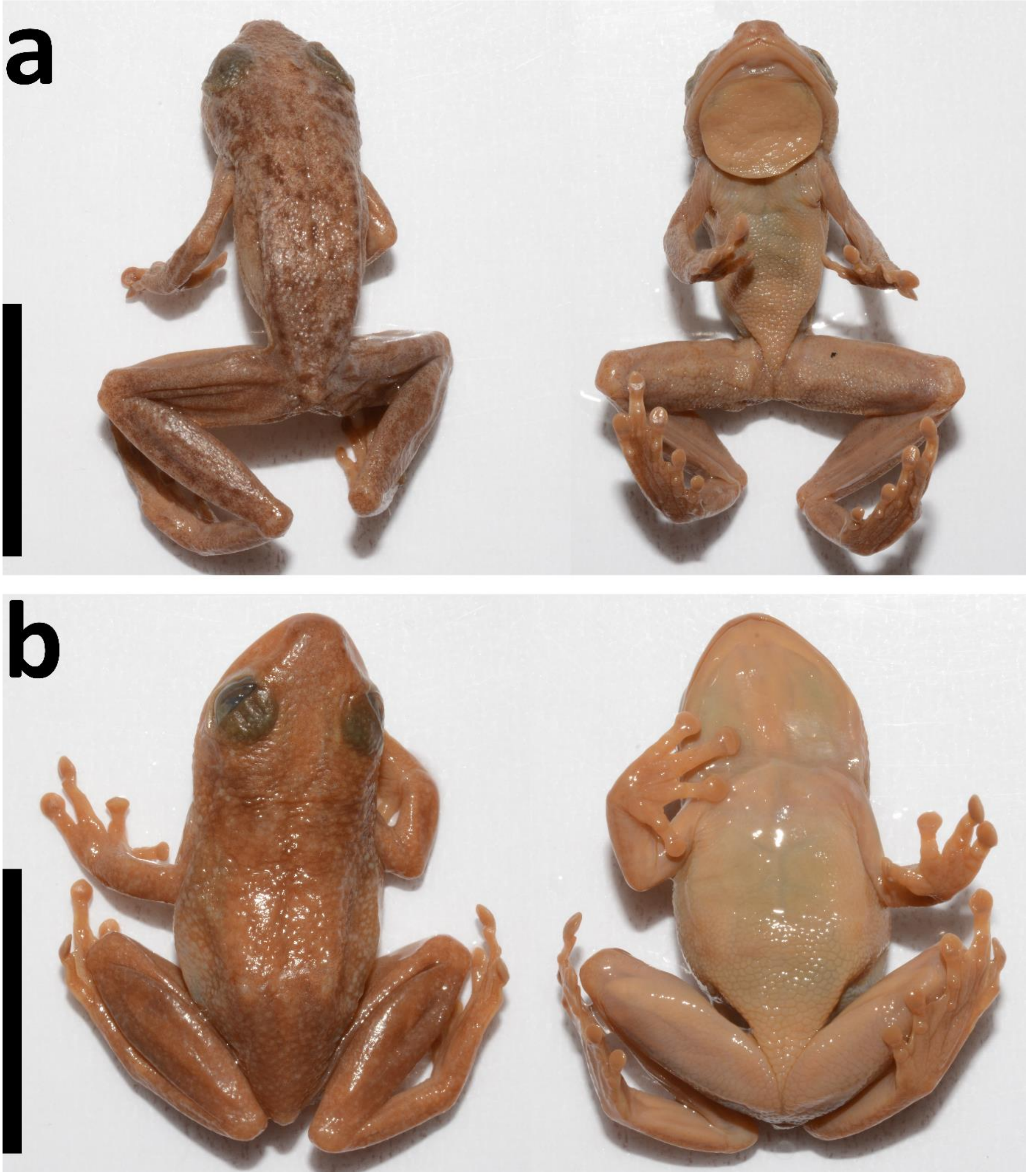

Figure S1. Holotype and allotype of *Hyperolius robustus* Laurent, 1979. (a) Holotype RMCA 79-24-B-4, male; (b) allotype RMCA 79-24-B-5, female. Scale = 20 mm. Photos by V. G.

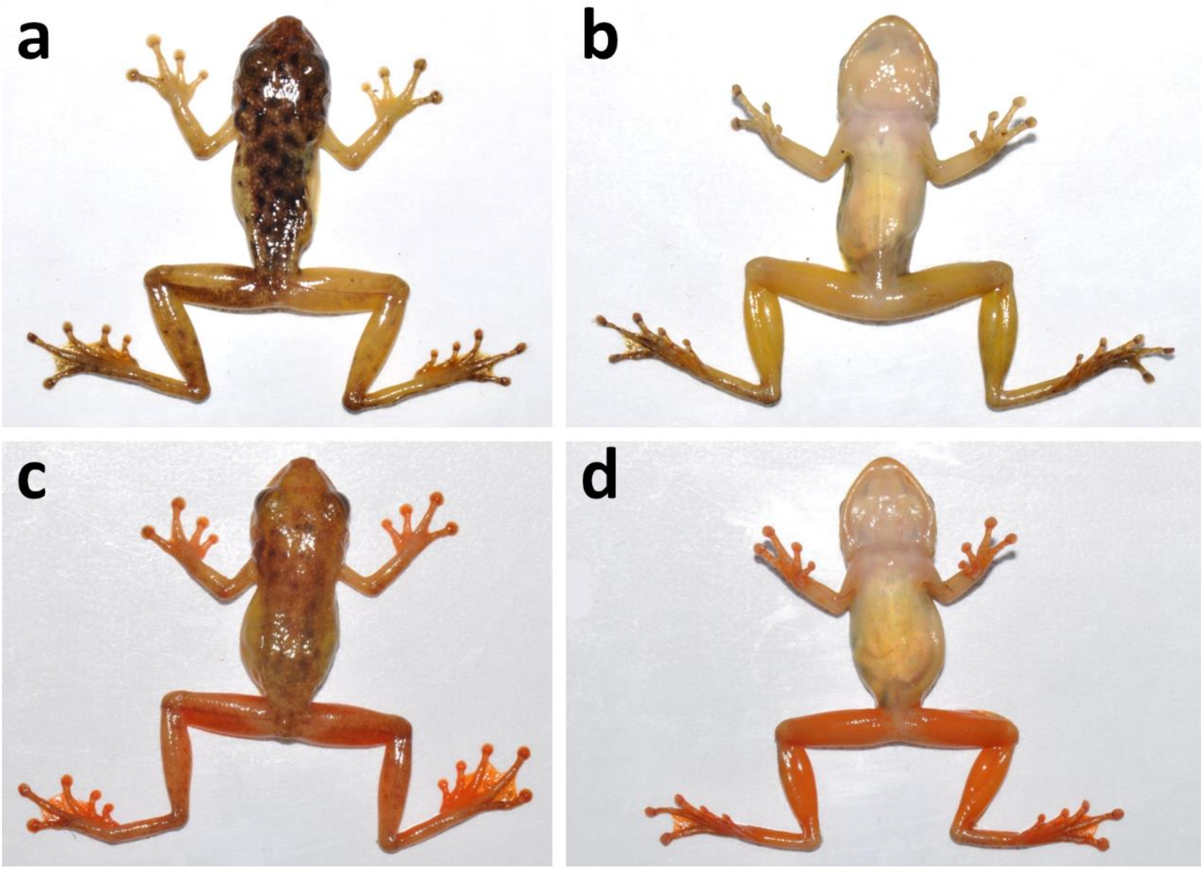

Figure S2. Sexual dichromatism in *Congolius robustus* gen. et comb. n. (a, b) Adult male IVB-H-CD18-301 and (c, d) adult female IVB-H-CD18-302 from the Kokolopori Bonobo Nature Reserve, Tshuapa, Democratic Republic of the Congo (DRC). Photos by V. G.

Table S1. Main material examined. Asterisk denotes specimens of *Congolius robustus* gen. et comb. n. included in phylogenetic analysis. “Morph” stands for external morphometric analyses; (+) denotes μCT scanned specimens that were not included in PCA of the cranial shape; CAR = Central African Republic.

Taxon	Catalogue No.	Sex	Locality	GPS °N	GPS °E	μCT	Morph
Acanthixalus spinosus	CAS 153800	female	Sangmelima, Foulassi, Ngam, Sud Region, Cameroon	2.9845	11.9612	(+)	-
Callixalus pictus	CAS 145260	male	Itombwe Highlands, upper Luvubu River, Kivu Prov., DRC, 2600 m	-3.2162	28.8612	(+)	-
Chrysobatrachus cupreonitens	CAS 145263	male	Itombwe Highlands, upper Luvubu River, Kivu Prov., DRC, 2600 m	-3.2162	28.8612	(+)	-
Congolius robustus gen. et comb. n.	IVB-H-CD18-120	male	Yetee, Loto stream, Kokolopori, Tshuapa, DRC, 460 m	0.4080	22.9284	-	+
*Congolius robustus**	IVB-H-CD18-255	male	Yalokole, Iyoto stream, Kokolopori, Tshuapa, DRC, 400 m	0.2136	22.8949	-	+
Congolius robustus	NMP-P6V 76086/1	male	Yalokole, Iyoto stream, Kokolopori, Tshuapa, DRC, 400 m	0.2136	22.8949	-	+
Congolius robustus	NMP-P6V 76086/2	male	Yalokole, Iyoto stream, Kokolopori, Tshuapa, DRC, 400 m	0.2136	22.8949	-	+
Congolius robustus	NMP-P6V 76086/4	male	Yalokole, Iyoto stream, Kokolopori, Tshuapa, DRC, 400 m	0.2136	22.8949	-	+
Congolius robustus	NMP-P6V 76086/5	male	Yalokole, Iyoto stream, Kokolopori, Tshuapa, DRC, 400 m	0.2136	22.8949	-	+
Congolius robustus	NMP-P6V 76086/8	male	Yalokole, Iyoto stream, Kokolopori,Tshuapa, DRC, 400 m	0.2136	22.8949	-	+
Congolius robustus	NMP-P6V 76086/9	male	Yalokole, Iyoto stream, Kokolopori, Tshuapa, DRC, 400 m	0.2136	22.8949	-	+
Congolius robustus	NMP-P6V 76088	male	Yambimbo, Sondo stream, Kokolopori, Tshuapa, DRC, 420 m	0.2235	22.8627	-	+
*Congolius robustus**	NMP-P6V 76089	female	Mombongo, Luende stream, Tshopo, DRC	1.64	23.15	-	-
Congolius robustus	ZMUC-R.771175	male	Kokolopori, Tshuapa, DRC	0.25	22.87	+	+
*Congolius robustus**	ZMUC-R.771176	female	Kokolopori, Tshuapa, DRC	0.25	22.87	(+)	-
Cryptothylax greshoffii	IVB-H-CD15-162	male	Lopori River, Matama, Lobeke swamp, Mongala, DRC, 365 m	1.6366	21.4172	-	+
Cryptothylax greshoffii	IVB-H-CD15-169	male	Lopori River, Matama, Lobeke swamp, Mongala, DRC, 365 m	1.6366	21.4172	+	+
Cryptothylax greshoffii	IVB-H-CD15-170	male	Lopori River, Matama, Lobeke swamp, Mongala, DRC, 365 m	1.6366	21.4172	-	+
Cryptothylax greshoffii	NMP-P6V 76074/1	male	Sangha Lodge, swamp, Sangha-Mbaéré, Dzanga-Sangha NP, CAR, 380 m	2.99	16.23	-	+
Cryptothylax greshoffii	NMP-P6V 76075/1	male	Sangha Lodge, forest pond, Sangha-Mbaéré, Dzanga-Sangha NP, CAR, 380 m	2.9852	16.2327	-	+
Cryptothylax greshoffii	NMP-P6V 76075/2	male	Sangha Lodge, forest pond, Sangha-Mbaéré, Dzanga-Sangha NP, CAR, 380 m	2.9852	16.2327	-	+
Cryptothylax greshoffii	NMP-P6V 76076/1	male	Mosite, pond, Tshopo, DRC, 420 m	0.9594	23.5549	-	+
Cryptothylax greshoffii	NMP-P6V 76076/2	male	Mosite, pond, Tshopo, DRC, 420 m	0.9594	23.5549	-	+

Taxon	Catalogue No.	Sex	Locality	GPS		µCT	Morph
				°N	°E		
Cryptothylax greshoffii	NMP-P6V 76077/7	male	Yetee, Loto stream, Kokolopori, Tshuapa, DRC, 460 m	0.408	22.9284	-	+
Cryptothylax greshoffii	NMP-P6V 76078/1	male	Yalofili, Bisolo stream, Tshuapa, DRC, 400 m	0.2105	22.8186	-	+
Hyperolius balfouri	IVB-H-CD15-061	male	Lisala, swamp, Mongala, DRC, 348 m	2.1503	21.5285	+	-
Hyperolius balfouri	NMP-P6V 76079/1	male	Baboungue, Sangha-Mbaéré, Dzanga-Sangha NP, CAR, 550 m	3.2815	16.1854	-	+
Hyperolius balfouri	NMP-P6V 76079/2	male	Baboungue, Sangha-Mbaéré, Dzanga-Sangha NP, CAR, 550 m	3.2815	16.1854	-	+
Hyperolius balfouri	NMP-P6V 76080/1	male	Baboungue, Sangha-Mbaéré, Dzanga-Sangha NP, CAR, 550 m	3.2815	16.1854	-	+
Hyperolius balfouri	NMP-P6V 76080/3	male	Baboungue, Sangha-Mbaéré, Dzanga-Sangha NP, CAR, 550 m	3.2815	16.1854	-	+
Hyperolius balfouri	NMP-P6V 76080/4	male	Baboungue, Sangha-Mbaéré, Dzanga-Sangha NP, CAR, 550 m	3.2815	16.1854	-	+
Hyperolius balfouri	NMP-P6V 76081/2	male	Monasau, by road, Sangha-Mbaéré, Dzanga-Sangha NP, CAR, 530 m	3.2487	16.2526	-	+
Hyperolius balfouri	NMP-P6V 76081/1	male	Monasau, by road, Sangha-Mbaéré, Dzanga-Sangha NP, CAR, 530 m	3.2487	16.2526	-	+
Hyperolius balfouri	NMP-P6V 76081/2	male	Monasau, by road, Sangha-Mbaéré, Dzanga-Sangha NP, CAR, 530 m	3.2487	16.2526	-	+
Hyperolius balfouri	NMP-P6V 76081/3	male	Monasau, by road, Sangha-Mbaéré, Dzanga-Sangha NP, CAR, 530 m	3.2487	16.2526	-	+
Hyperolius balfouri	NMP-P6V 76081/5	male	Monasau, by road, Sangha-Mbaéré, Dzanga-Sangha NP, CAR, 530 m	3.2487	16.2526	-	+
Hyperolius cinnamomeoventris complex	IVB-H-CD18-092	male	Yetee, spring, Kokolopori, Tshuapa, DRC, 470 m	0.4033	22.9302	-	+
Hyperolius cinnamomeoventris complex	IVB-H-CD18-107	male	Yetee, Loto stream, Kokolopori, Tshuapa, DRC, 460 m	0.408	22.9284	-	+
Hyperolius cinnamomeoventris complex	IVB-H-CD18-129	male	Yetee, Loto stream, Kokolopori, Tshuapa, DRC, 460 m	0.408	22.9284	-	+
Hyperolius cinnamomeoventris complex	IVB-H-CD18-130	male	Yetee, Loto stream, Kokolopori, Tshuapa, DRC, 460 m	0.408	22.9284	-	+
Hyperolius cinnamomeoventris complex	IVB-H-CD18-131	male	Yetee, Loto stream, Kokolopori, Tshuapa, DRC, 460 m	0.408	22.9284	-	+
Hyperolius cinnamomeoventris complex	IVB-H-CD18-132	male	Yetee, Loto stream, Kokolopori, Tshuapa, DRC, 460 m	0.408	22.9284	-	+
Hyperolius cinnamomeoventris complex	IVB-H-CD18-133	male	Yetee, Loto stream, Kokolopori, Tshuapa, DRC, 460 m	0.408	22.9284	-	+
Hyperolius cinnamomeoventris complex	IVB-H-CD18-134	male	Yetee, Loto stream, Kokolopori, Tshuapa, DRC, 460 m	0.408	22.9284	-	+
Hyperolius cinnamomeoventris complex	IVB-H-CD18-357	male	Yambimbo, Sondo stream, Kokolopori, Tshuapa, DRC, 420 m	0.2255	22.8616	-	+
Hyperolius cinnamomeoventris complex	IVB-H-CD18-358	male	Yambimbo, Sondo stream, Kokolopori, Tshuapa, DRC, 430 m	0.2255	22.8616	-	+

Taxon	Catalogue No.	Sex	Locality	GPS		μCT	Morph
				°N	°E		
Hyperolius phantasticus	IVB-H-CD18-192	male	Yalokole, Iyoto stream, Kokolopori, Tshuapa, DRC, 400 m	0.2136	22.8949	-	+
Hyperolius phantasticus	IVB-H-CD18-202	male	Yalofili, Bisolo stream, Kokolopori, Tshuapa, DRC, 400 m	0.2105	22.8186	-	+
Hyperolius phantasticus	IVB-H-CD18-492	male	Yambimbo, Sondo stream, Kokolopori, Tshuapa, DRC, 420 m	0.2269	22.8621	-	+
Hyperolius phantasticus	IVB-H-CD18-493	male	Yambimbo, Sondo stream, Kokolopori, Tshuapa, DRC, 420 m	0.2269	22.8621	-	+
Hyperolius phantasticus	IVB-H-CD18-508	male	Yambimbo, Sondo stream, Kokolopori, Tshuapa, DRC, 420 m	0.2269	22.8621	-	+
Hyperolius phantasticus	IVB-H-CD18-518	male	Yambimbo, Sondo stream, Kokolopori, Tshuapa, DRC, 420 m	0.2269	22.8621	-	+
Hyperolius phantasticus	IVB-H-CD18-519	male	Yambimbo, Sondo stream, Kokolopori, Tshuapa, DRC, 420 m	0.2269	22.8621	-	+
Hyperolius phantasticus	IVB-H-CD18-523	male	Yambimbo, Sondo stream, Kokolopori, Tshuapa, DRC, 420 m	0.2269	22.8621	-	+
Hyperolius phantasticus	IVB-H-CD18-526	male	Yambimbo, Sondo stream, Kokolopori, Tshuapa, DRC, 420 m	0.2269	22.8621	-	+
Hyperolius phantasticus	IVB-H-CD18-528	male	Yambimbo, Sondo stream, Kokolopori, Tshuapa, DRC, 420 m	0.2269	22.8621	-	+
Hyperolius tuberculatus complex	NMP-P6V 76082/1	male	Mafamba, camp, Pool, Rep. Congo, 282 m	-4.2789	16.1973	-	+
Hyperolius tuberculatus complex	NMP-P6V 76082/4	male	Mafamba, camp, Pool, Rep. Congo, 282 m	-4.2789	16.1973	-	+
Hyperolius tuberculatus complex	NMP-P6V 76082/5	male	Mafamba, camp, Pool, Rep. Congo, 282 m	-4.2789	16.1973	-	+
Hyperolius tuberculatus complex	NMP-P6V 76082/7	male	Mafamba, camp, Pool, Rep. Congo, 282 m	-4.2789	16.1973	-	+
Hyperolius tuberculatus complex	NMP-P6V 76082/8	male	Mafamba, camp, Pool, Rep. Congo, 282 m	-4.2789	16.1973	-	+
Hyperolius tuberculatus complex	NMP-P6V 76082/10	male	Mafamba, camp, Pool, Rep. Congo, 282 m	-4.2789	16.1973	-	+
Hyperolius tuberculatus complex	NMP-P6V 76082/11	male	Mafamba, camp, Pool, Rep. Congo, 282 m	-4.2789	16.1973	-	+
Hyperolius tuberculatus complex	NMP-P6V 76082/14	male	Mafamba, camp, Pool, Rep. Congo, 282 m	-4.2789	16.1973	-	+
Hyperolius tuberculatus complex	NMP-P6V 76083	male	Mafamba, site 1, Pool, Rep. Congo, 274 m	-4.2789	16.1967	-	+
Hyperolius tuberculatus complex	NMP-P6V 76084	male	Mafamba, site 2, Pool, Rep. Congo, 271 m	-4.2789	16.1961	-	+
Hyperolius tuberculatus complex	IVB-H-CD15-010	male	Lisala, Congo River, Bapoto-Mongo, Mongala, DRC, 345 m	2.1511	21.535	+	-

Appendix S1. Additional material examined.

Afrixalus equatorialis: NMP-P6V 76073/1 (Yalofili, Bisolo stream, Kokolopori, Tshuapa, DRC; 0.21°N 22.82°E).

Callixalus pictus: RMCA B.105145 (holotype; Lutsiro, 2600 m, Kisenyi Territory, Rwanda); CAS 104963, 104965–104967, 145261 (Upper Luvubu River, 2600–2800 m, Itombwe Highlands, Uvira Territory, South Kivu, DRC); MCZ A-64705–64714 (Upper Luvubu River, 2600 m, Itombwe Highlands, Uvira Territory, South Kivu, DRC).

Chrysobatrachus cupreonitens: RMCA B.109970 (holotype; Kitadjabukwe River 2800–2850 m, Uvira Territory, South Kivu, DRC); CAS 104968–104969, 104971–104972 (paratypes; Upper Kilungutwe swamp, 2650 m, Itombwe Highlands, Uvira Territory, South Kivu, DRC); MCZ A-64730–64739 (paratypes; Upper Kilungutwe swamps, 2650 m, Itombwe Highlands, Uvira Territory, South Kivu, DRC).

Congolius robustus gen. et comb. n.: RMCA 79-24-B-4 (holotype; Gembe, Koiteko swamp, Sankuru, DRC; -3.48°N 23.43°E), 79-24-B-5 (allotype; same locality as holotype), B.61800 (Makaw, Kasai River, Maï-Ndombe, DRC; -3.47°N 18.30°E); NMP-P6V 76087/2 (Yalokole, Luo River, swamp, Kokolopori, Tshuapa, DRC; 0.21°N 22.89°E), IVB-H-CD18-301–302 (Kima Mpela, stream, Kokolopori, Tshuapa, DRC; 0.17°N 22.93°E); ZMUC-R.079697 (Monkoto, Tshuapa, DRC ; -1.58°N 20.67°E), R.771151 (Landu River, Lodja, Sankuru, DRC; -3.25°N 23.52°E), R.771174, R.771177–771179 (Kokolopori, Tshuapa, DRC; 0.25°N 22.87°E).

Cryptothylax minutus: RMCA B.117082 (holotype; Mabali, Bikoro, Lake Tumba, Équateur, DRC), specimen # 55 from series B.117083–117087 (no individual acc. no., allotype; same locality as holotype).

Hyperolius balfouri: IVB-H-CD15-062 (Lisala, swamp, Mongala, Equateur, DRC; 2.15°N 21.53°E).

H. cf. *adspersus*: IVB-H-CD15-139 (Bosolo, spring, Mongala, Equateur, DRC; 1.75°N 21.42°E).

H. sankuruensis: RMCA 79-24-B-1 (holotype; Omaniundu, Sankuru, DRC; -3.35°N 23.27°E), 79-24-B-2 (allotype; same locality as holotype); ZMUC-R.771205 (Mabali, Lake Tumba, old IRSAC station, Équateur, DRC; -0.88°N 18.13°E).

Opisthothylax immaculatus: NMP-P6V 76090/1 (Mindjong, Mbemba forest, site2, Sembé, Sangha, Rep. Congo; 1.49°N 14.37°E).

Phlyctimantis verrucosus: NMP-P6V 76085/1 (Yasaka, Kokolopori, Tshuapa, DRC; 0.24°N 22.87°E).

Appendix S2. Distribution records of *Congolius robustus* gen. et comb. n.

Based on literature, museum materials, and own unpublished data, we provide a list of distribution records of *Congolius robustus* gen. et comb. n., all in present-day DRC (coordinates are approximate; former provinces in parentheses; see Fig. 1a):
Gembe, Koiteko Swamp, Lodja Territory, type locality, -3.48°N 23.43°E, Sankuru (Kasaï-Oriental), Laurent (1979);
Musii River, Lodja Territory, -3.56°N, 23.65°E, Sankuru (Kasaï-Oriental), Laurent (1979);
Onema, Lodja Territory, -3.72°N 23.92°E, Sankuru (Kasaï-Oriental), Laurent (1979);
Omaniundu, Lodja Territory , -3.35°N 23.27°E, Sankuru (Kasaï-Oriental), Laurent (1979);
Lomami, Lomela Territory, -1.92°N 22.70°E, Sankuru (Kasaï-Oriental), Laurent (1979);
Landu River, Lodja, -3.25°N 23.52°E, Sankuru (Kasaï-Oriental), single specimen ZMUC-R.771151 (on exchange from RMCA);
Makaw, Kasai River, -3.47°N 18.30°E, Maï-Ndombe (Bandundu), single specimen RMCA B.61800, collected by E. Jans in 1958;
LuiKotale, Salonga National Park, -2.88°N 20.41°E, Maï-Ndombe (Bandundu), recorded by J. Kielgast in 2008 (pers. comm.);
Monkoto, -1.58°N 20.67°E, Tshuapa (Équateur), Schiøtz (1999, 2006);
Kokolopori Bonobo Nature Reserve, 0.25°N 22.87°E, Tshuapa (Équateur), Schiøtz (2006; the locality was given incorrectly as south of the Equator), several sites within the reserve (own unpublished data; see Supplementary Table S1 for details on investigated material);
Mombongo, Luende stream, 1.64°N 23.15°E, Tshopo (Orientale), this study;
Yawaka, Bechuchuu, 0.51°N 23.15°E, Tshopo (Orientale), this study.

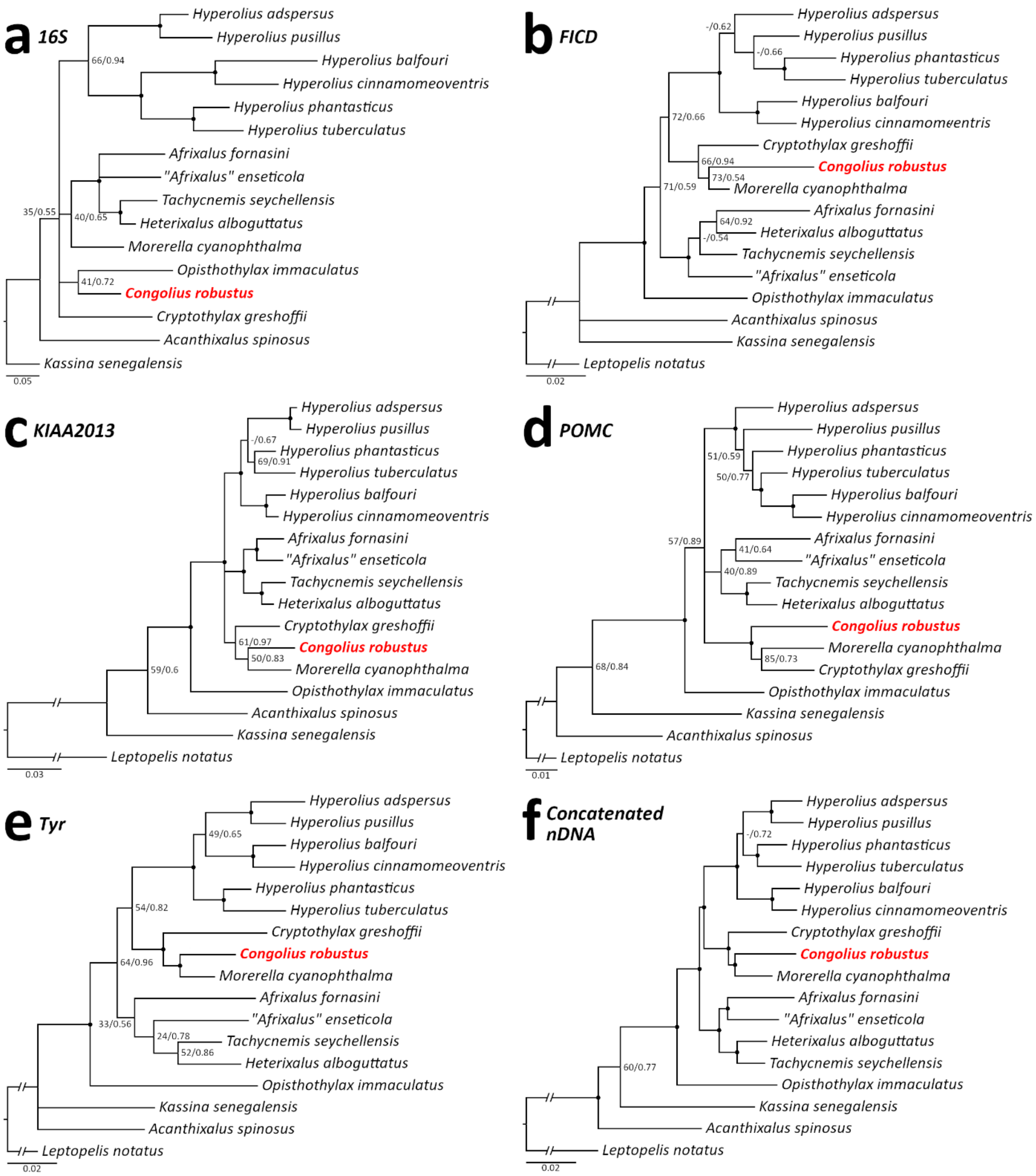

Figure S3. Bayesian phylogenetic trees of (a–e) individual genetic markers, and (f) concatenated data set based on four nuclear markers. (a) Mitochondrial *16S rRNA*, (b) *FICD*, (c) *KIAA2013*, (d) *POMC*, (e) *Tyr*, and (f) concatenated nuclear data set. Dots at nodes represent high supports (ML bootstrap ≥ 70/Bayesian posterior probabilities ≥ 0.95), and numbers show support values for nodes with lower support. GenBank accession numbers are provided in Supplementary Table S5.

Appendix S3. Resources for comparative diagnosis of *Congolius* gen. n. with other genera of the family Hyperoliidae.

Morphological characters were taken from Amiet (2000, *Alexteroon*; 2012), Channing *et al*. (2013, *Hyperolius*), Drewes (1984, *Heterixalus*, *Paracassina*, *Semnodactylus*, and other genera), Largen (1975, *Kassina*), Laurent (1940, *Kassinula*; 1950, *Callixalus*; 1951, *Chrysobatrachus*; 1964, *Callixalus, Chrysobatrachus*; 1976, *Cryptothylax, Phlyctimantis, Kassina*; 1982, *Afrixalus*), Laurent & Combaz (1950, *Cryptothylax, Phlyctimantis*), Liem (1970), Nussbaum & Wu (1995, *Tachycnemis*), Perret (1988, *Alexteroon*, *Arlequinus*), Rödel *et al*. (2003, *Acanthixalus*; 2009, *Morerella*), Schiøtz (1975; 1999; 2006, *Hyperolius*; 2007), and own unpublished data. See Supplementary Appendix S4 for references.

Table S2. Uncorrected *p*-distances based on a fragment of the *16S rRNA* gene. Asterisk denotes specimens of *Congolius robustus* gen. et comb. n. from the same locality (Kokolopori Bonobo Nature Reserve, DRC).

	Species	GenBank acc. numbers	1	2	3	4	5	6	7	8	9	10	11	12	13	14	15	16	17	18
1	*Congolius robustus* gen. et comb. n.* ZMUC-R.771176	MW626928	-																	
2	*Congolius robustus** IVB-H-CD18-255	MW626930	0.0	-																
3	*Congolius robustus* NMP-P6V 76089	MW626929	0.4	0.4	-															
4	*Morerella cyanophthalma*	MK498163	11.8	11.6	11.6	-														
5	*Cryptothylax greshoffii*	MK509715	15.0	15.0	13.6	13.8	-													
6	*Hyperolius adspersus*	MK509502	17.8	17.1	17.5	17.2	18.8	-												
7	*Hyperolius pusillus*	MK509496	18.0	16.8	17.3	17.2	20.2	8.5	-											
8	*Hyperolius balfouri*	MK509719	20.6	19.8	20.2	20.3	19.9	19.5	19.0	-										
9	*Hyperolius cinnamomeoventris*	MK509566	20.6	20.2	20.6	20.7	19.7	19.1	17.9	14.5	-									
10	*Hyperolius phantasticus*	MK509672	19.2	19.2	19.6	15.8	19.5	17.1	17.8	19.0	17.9	-								
11	*Hyperolius tuberculatus*	MK509553	20.2	19.8	20.2	16.0	19.9	18.6	17.6	20.0	19.2	9.0	-							
12	*Afrixalus fornasini*	MK509484	14.1	14.1	14.5	13.5	16.9	17.1	18.2	21.4	19.8	18.8	19.1	-						
13	"*Afrixalus*" *enseticola*	MK509689	13.6	13.5	13.5	11.9	15.4	17.0	17.9	21.3	20.1	17.8	16.3	11.7	-					
14	*Heterixalus alboguttatus*	MK509492	13.6	13.5	13.9	9.8	14.4	16.4	16.9	21.6	20.5	16.7	17.3	11.0	9.7	-				
15	*Tachycnemis seychellensis*	MK509675	13.6	15.4	15.9	11.4	14.8	19.5	19.9	22.8	19.8	18.3	17.9	10.8	10.2	6.8	-			
16	*Opisthothylax immaculatus*	MK509570	12.8	12.0	12.4	13.2	15.1	17.6	18.1	20.9	18.6	18.9	19.0	15.1	12.8	13.4	14.6	-		
17	*Acanthixalus spinosus*	MK509618	16.5	16.1	16.1	11.9	18.2	19.7	19.7	21.7	20.7	19.0	19.2	16.9	18.0	15.3	17.8	18.4	-	
18	*Kassina senegalensis*	MK509663	13.6	13.1	13.5	13.6	13.7	17.5	18.6	20.0	19.4	17.7	19.1	14.5	15.9	14.1	15.5	13.2	15.2	-

Table S3. Factor loadings of the first three principal components of PCA of body shape.
Values of the most contributing variables in bold.

Component	HW	HDL	IOD	ED	ENL	IND	SL	HL	RL	HaL	DW	FL	TL	FoL	MANOVA
PC1 (31.7%)	-0.24	**-1.12**	0.86	0.87	**-1.11**	**1.14**	**-1.14**	0.49	0.37	-0.44	0.76	0.25	0.72	-0.87	$F_{5,15}$ = 57.07
PC2 (20.3%)	**1.00**	0.15	0.74	0.18	-0.38	0.68	0.02	**-1.03**	**-0.94**	0.36	0.15	-0.78	**-0.94**	-0.22	*p* = <2.2e-16
PC3 (12.9%)	**-0.68**	0.25	0.22	0.59	**0.69**	-0.12	0.47	-0.41	-0.40	**-0.98**	-0.01	-0.30	0.17	**-0.83**	

Congolius robustus gen. et comb. n.
ZMUC-R.771175

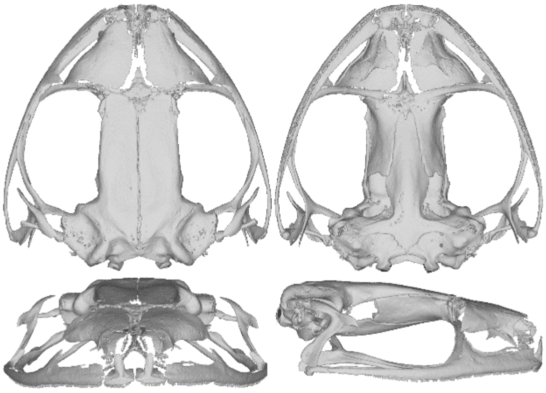

Congolius robustus gen. et comb. n.
ZMUC-R.771176

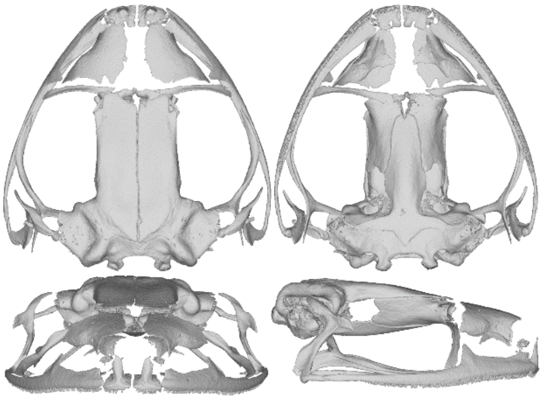

Hyperolius balfouri
IVB-H-CD15-061

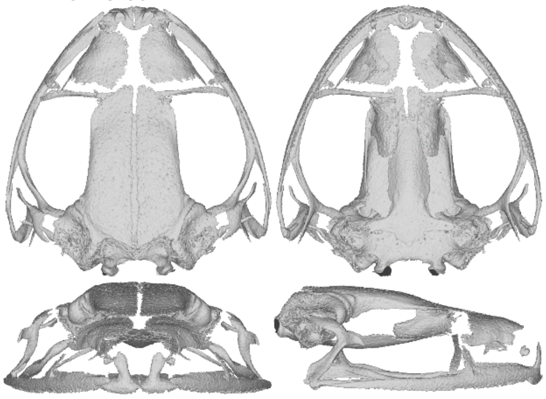

Hyperolius tuberculatus
IVB-H-CD15-010

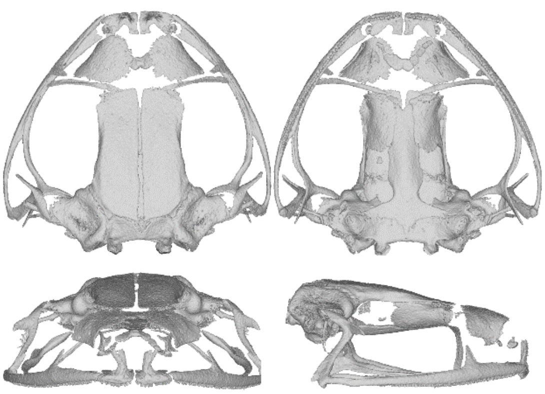

Cryptothylax greshoffii
IVB-H-CD15-169

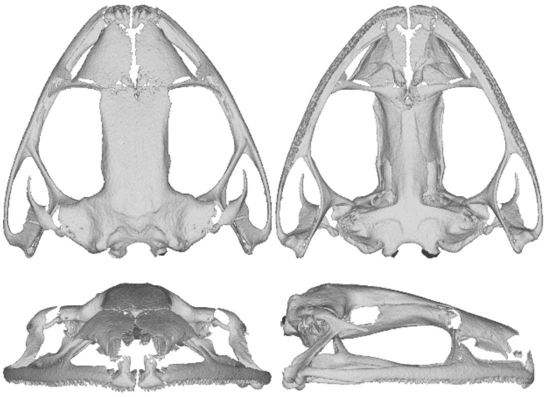

Chrysobatrachus cupreonitens
CAS 145263

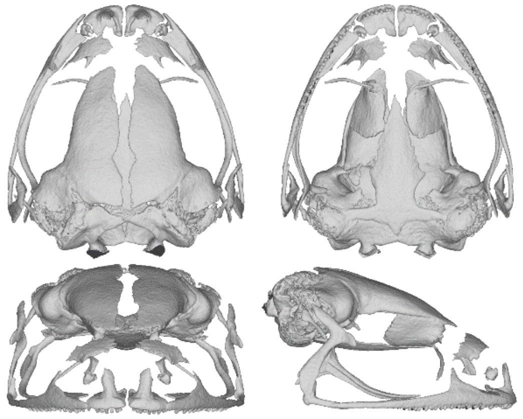

Callixalus pictus
CAS 145260

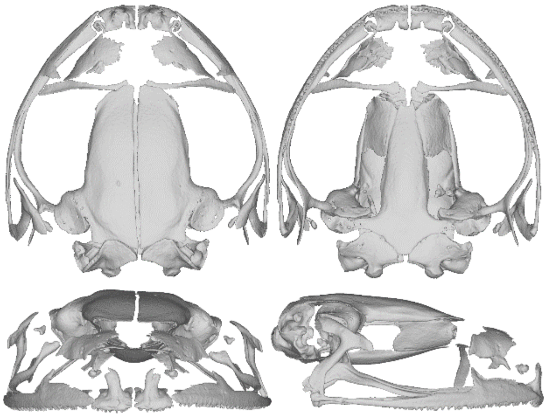

Acanthixalus spinosus
CAS 153800

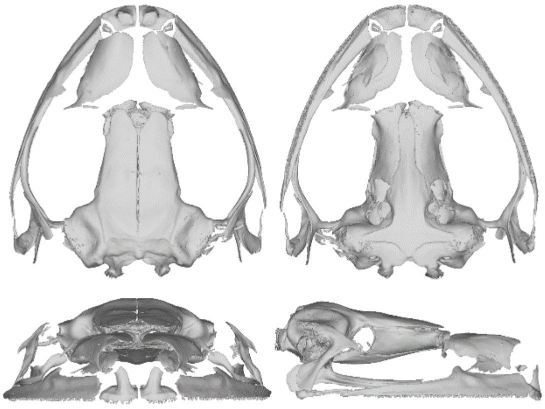

Figure S4. Cranial morphology of all examined specimens. From top left: *Congolius robustus* gen. et comb. n. (ZMUC-R.771175, male), *C. robustus* (ZMUC-R.771176, female), *Hyperolius balfouri* (IVB-H-CD15-061, male), *H.* cf. *tuberculatus* (IVB-H-CD15-010, male), *Cryptothylax greshoffii* (IVB-H-CD15-169, male), *Chrysobatrachus cupreonitens* (CAS 145263, male), *Callixalus pictus* (CAS 145260, male), and *Acanthixalus spinosus* (CAS 153800, female). Reconstructions in dorsal, ventral, frontal, and lateral views are based on high-resolution X-ray microcomputed tomography. Skull views are scaled to a similar size among the specimens.

Table S4. Factor scores of the first two principal components of PCA of cranial shape. Taxa in bold are similar in the shape of their skulls.

Component	*Hyperolius tuberculatus*	***Hyperolius balfouri***	***Congolius robustus*** gen. et comb. n.	*Cryptothylax greshoffii*
PC1 (65.1%)	-0.064	**-0.018**	**0.007**	0.076
PC2 (28.5%)	0.038	**-0.031**	**-0.036**	0.028

Table S5. Taxa and samples included in phylogenetic analyses with GenBank accession numbers. Asterisk denotes outgroups.

Species	*16S*	*FICD*	*KIAA2013*	*POMC*	*Tyr*
**Acanthixalus spinosus*	MK509618	MK498693	MK498898	MK499174	MK498413
Afrixalus enseticola	MK509689	MK498520	MK498923	MK498959	MK498374
Afrixalus fornasini	MK509484	MK498507	MK498804	MK499057	MK498390
Congolius robustus gen. et. comb. n. ZMUC-R.771176	MW626928	MW626916	MW626919	MW626922	MW626925
Congolius robustus NMP-P6V 76089	MW626929	MW626917	MW626920	MW626923	MW626926
Congolius robustus IVB-H-CD18-255	MW626930	MW626918	MW626921	MW626924	MW626927
Cryptothylax greshoffii	MK509715	MK498689	MK498705	MK498956	MK498434
Heterixalus alboguttatus	MK509492	MK498523	MK498893	MK499154	MK498371
Hyperolius adspersus	MK509502	MK498632	MK498931	MK498992	MK498264
Hyperolius balfouri	MK509719	MK498481	MK498869	MK499101	MK498032
Hyperolius cinnamomeoventris	MK509566	MK498473	MK498811	MK499132	MK498272
Hyperolius phantasticus	MK509672	MK498677	MK498794	MK498998	MK498265
Hyperolius pusillus	MK509496	MK498603	MK498887	MK499047	MK498225
Hyperolius tuberculatus	MK509553	MK498635	MK498878	MK499111	MK498206
**Kassina senegalensis*	MK509663	MK498621	MK498940	MK499186	MK498427
**Leptopelis notatus*	-	KX492679	KX492732	KX492782	KX492876
Morerella cyanophthalma	MK498163	MK498608	MK498915	MK499159	MK498411
Opisthothylax immaculatus	MK509570	MK498629	MK498946	MK499171	MK498430
Tachycnemis seychellensis	MK509675	MK498585	MK498924	MK499156	MK498396

Table S6. Primers used to amplify selected markers.

Marker	Primer	Sequence (5'–3')	Source
16S	16SL1	CGC CTG TTT AAC AAA AAC AT	Palumbi *et al*. 1991 (modified)
16S	16SH1	CCG GTC TGA ACT CAG ATC ACG T	Palumbi *et al*. 1991
FICD	FICD F1	CCK CTN GTN GAR ATH GAY CA	Shen *et al*. 2013
FICD	FICD R1	TYT CNG TRC AYT TNG CDA TRA A	Shen *et al*. 2013
FICD	FICD F2	AGG GTT TTC CCA GTX ACG ACT ACT AYC AYC AYA THT AYC AYA C	Shen *et al*. 2013
FICD	FICD R2	AGA TAA CAA TTT CAC ACA GGA ARG GCC KVA CRT CNC CYT CRT T	Shen *et al*. 2013
KIAA2013	KIAA2013 F1	CTS AAN TAY GCN GAY CAY TGY TT	Shen *et al*. 2013
KIAA2013	KIAA2013 R1	CCN GGN CCR CAR TAY TCR TTR TA	Shen *et al*. 2013
KIAA2013	KIAA2013 F2	AGG GTT TTC CCA GTC ACG ACA CYA TGC AYG CNG AGA AYY TGT GG	Shen *et al*. 2013
KIAA2013	KIAA2013 R2	AGA TAA CAA TTT CAC ACA GGG ANG CCA CNC TRA ACC ARA A	Shen *et al*. 2013
POMC	POMC-1	GAA TGT ATY AAA GMM TGC AAG ATG GWC CT	Wiens *et al*. 2005
POMC	POMC-7	TGG CAT TTT TGA AAA GAG TCA T	Smith *et al*. 2005
Tyr	Tyr 1C	GGC AGA GGA WCR TGC CAA GAT GT	Bossuyt & Milinkovitch 2000
Tyr	Tyr 1G	TGC TGG CRT CTC TCC ART CCC A	Bossuyt & Milinkovitch 2000

Table S7. Substitution models used in phylogenetic analyses.

Separate analysis	MrBayes and RAxML-NG			StarBEAST2		
	1st	2nd	3rd	1st	2nd	3rd
16S	GTR+I+G			-		
FICD	K80+I	F81+I	K80+G	K80+I	F81+I	K80+G
KIAA2013	K80+G		HKY+G	K80+G		HKY+G
POMC	HKY+I		K80+G	HKY+I		K80+G
Tyr	K80+I+G		HKY+G	K80+I+G		HKY+G
Concatenated nDNA data set	MrBayes and RAxML-NG			StarBEAST2		
	1st	2nd	3rd			
FICD	HKY+I+G	HKY+I	K80+G	-		
KIAA2013	K80	HKY+I	HKY+G	-		
POMC	HKY+I+G		K80+G	-		
Tyr	HKY+I+G		HKY+G	-		
Concatenated nDNA + *16S* dataset	MrBayes and RAxML-NG			StarBEAST2		
	1st	2nd	3rd			
16S	GTR+I+G			-		
FICD	HKY+I	K80+G	HKY+I+G	-		
KIAA2013	K80	HKY+I	HKY+G	-		
POMC	HKY+I+G		K80+G	-		
Tyr	HKY+I+G		HKY+G	-		

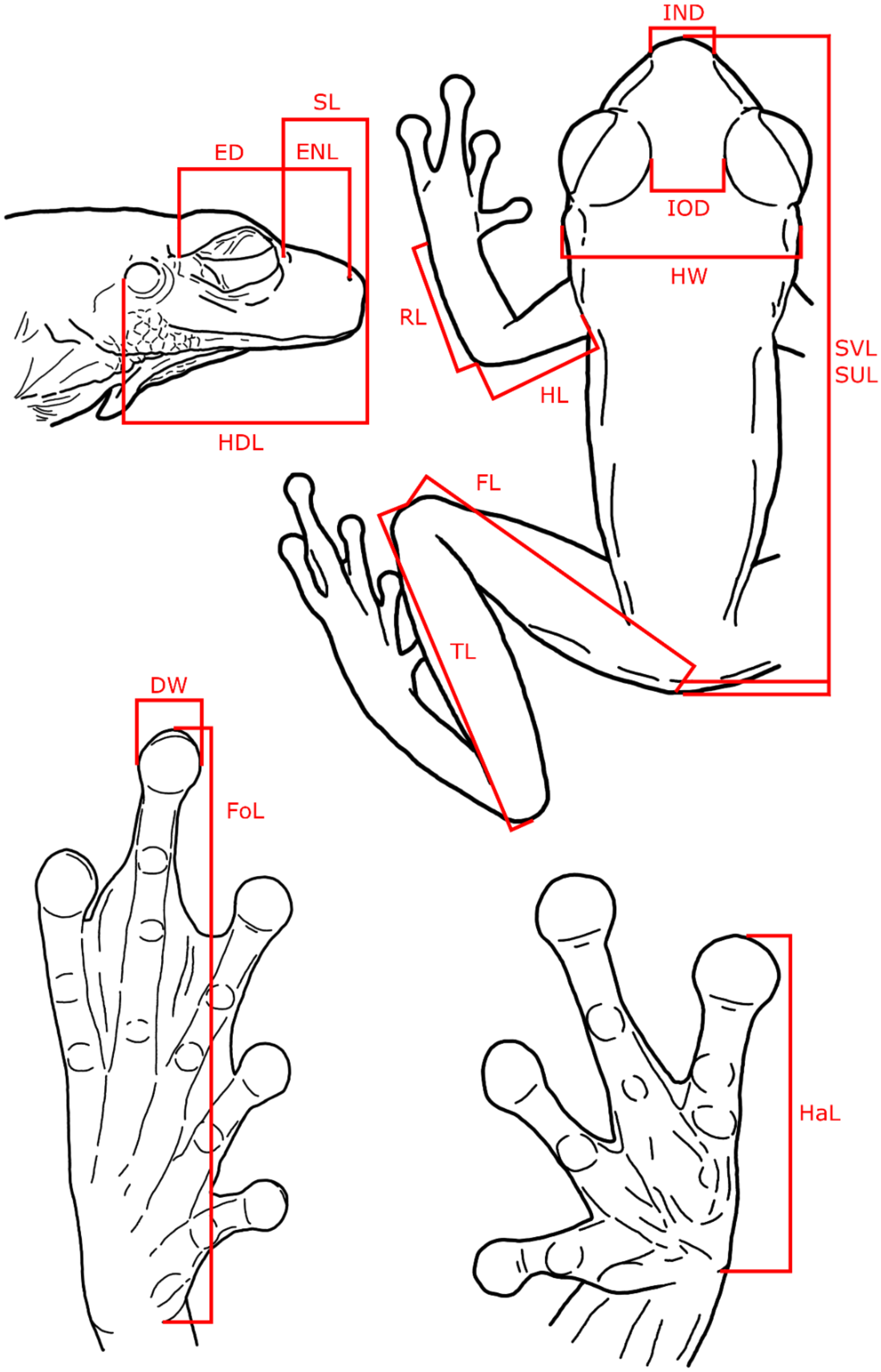

Figure S5. Visualization of 16 measured characters: snout-vent length (SVL), snout-urostyle length (SUL), head width (HW), head length (HDL), interorbital distance (IOD), eye diameter (ED), eye-nostril length (ENL), internarial distance (IND), snout length (SL), humerus length (HL), radioulna length (RL), hand length (HaL), femur length (FL), tibiofibula length (TL), foot length (FoL), disc width (DW). Illustration based on *Congolius robustus* gen. et comb. n. by T.N.

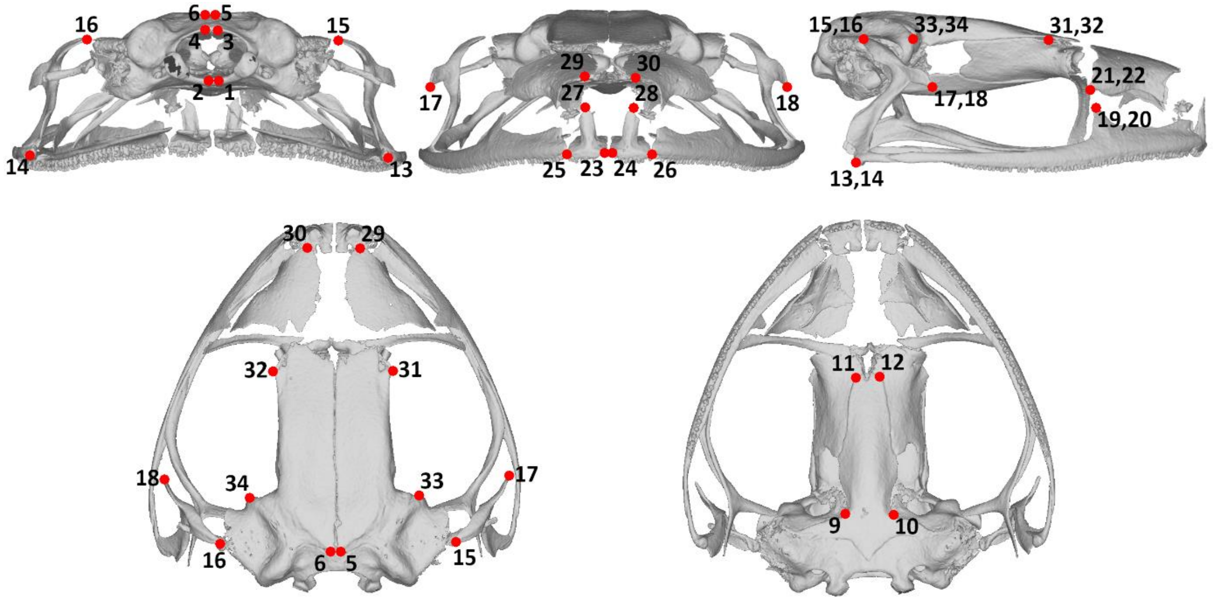

Figure S6. Visualization of 32 digitized landmarks. Adapted and modified from Paluh *et al*. (2020), displayed on a skull µCT scan of a female *Congolius robustus* gen. et comb. n. (ZMUC-R.771176). Views from top left: posterior, anterior, lateral, dorsal, and ventral.

Appendix S4. References used in Supplementary material in alphabetical order.

Amiet, J.-L. Les *Alexteroon* du Cameroun (Amphibia, Anura, Hyperoliidae). *Alytes* **17**, 125–164 (2000).

Amiet, J.-L. *Les rainettes du Cameroun (Amphibiens Anoures)* (J.-L. Amiet & La Nef des Livres, 2012).

Bossuyt, F. & Milinkovitch, M. C. Convergent adaptive radiations in Madagascan and Asian ranid frogs reveal covariation between larval and adult traits. *Proc. Natl. Acad. Sci. U.S.A.* **97**, 6585–6590 (2000).

Channing, A. & Rödel, M.-O. *Field Guide to the Frogs & Other Amphibians of Africa* (Struik Nature, 2019).

Channing, A. *et al*. Taxonomy of the super-cryptic *Hyperolius nasutus* group of long reed frogs of Africa (Anura: Hyperoliidae), with descriptions of six new species. *Zootaxa* **3620**, 301–350 (2013).

Drewes, R. C. A phylogenetic analysis of the Hyperoliidae (Anura): treefrogs of Africa, Madagascar, and the Seychelles Islands. *Occas. Pap. California Acad. Sci.* **139**, 1–70 (1984).

Largen, M. J. The status of the genus *Kassina* (Amphibia Anura Hyperoliidae) in Ethiopia. *Monit. Zool. Ital., Suppl. VI* **1**, 1–28 (1975).

Laurent, R. F. Description d'un Rhacophoride nouveau du Congo Belge (Batracien). *Rev. Zool. Bot. Afr.* **33**, 313–316 (1940).

Laurent, R. F. Diagnoses préliminaires de treize Batraciens nouveaux d'Afrique centrale. *Rev. Zool. Bot. Afr.* **44**, 1–18 (1950).

Laurent, R. F. Deux Reptiles et onze Batraciens nouveaux d'Afrique centrale. *Rev. Zool. Bot. Afr.* **44**, 360–381 (1951).

Laurent, R. F. Adaptive modifications in frogs of an isolated highland fauna in Central Africa. *Evolution* **18**, 458–467 (1964).

Laurent, R. F. Les genres *Cryptothylax, Phlyctimantis* et *Kassina* au Zaïre. *Ann. Mus. Congo Tervuren, Zool.* **213**, 1–67 (1976).

Laurent, R. F. Description de deux *Hyperolius* nouveaux du Sankuru (Zaire). *Rev. Zool. Bot. Afr.* **93**, 779–791 (1979).

Laurent, R. F. Le genre *Afrixalus* Laurent (Hyperoliidae) en Afrique centrale. *Ann. Mus. Congo Tervuren, Zool.* **235**, 1–58 (1982).

Laurent, R. F. & Combaz, J. Sur l'attribution générique de certains batraciens appartenant à la sous-famille des Hyperoliinae. *Rev. Zool. Bot. Afr.* **43**, 269–280 (1950).

Liem, S. S. The morphology, systematics, and evolution of the Old World treefrogs (Rhacophoridae and Hyperoliidae). *Fieldiana Zool.* **57**, 1–145 (1970).

Nussbaum, R. A. & Wu, S. H. Distribution, variation, and systematics of the Seychelles treefrog, *Tachycnemis seychellensis* (Amphibia: Anura: Hyperoliidae). *J. Zool.* **236**, 383–406 (1995).

Palumbi, S. *et al*. *The Simple Fool's Guide to PCR* (University of Hawaii, 1991).

Perret, J.-L. Sur quelques genres d'Hyperoliidae (Anura) restés en question. *Bull. Soc. Neuchâtel Sci. Nat.* **111**, 35–48 (1988).

Rödel, M.-O., Kosuch, J., Veith, M. & Ernst, R. First Record of the Genus *Acanthixalus* Laurent, 1944 from the Upper Guinean Rain Forest, West Africa, with the Description of a New Species. *J. Herpetol.* **37**, 43–52 (2003).

Rödel, M.-O. *et al*. A new tree-frog genus and species from Ivory Coast, West Africa (Amphibia: Anura: Hyperoliidae). *Zootaxa* **2044**, 23–45 (2009).

Schiøtz, A. *The Treefrogs of Eastern Africa* (Steenstrupia, 1975).

Schiøtz, A. *Treefrogs of Africa* (Edition Chimaira, 1999).

Schiøtz, A. Notes on the genus *Hyperolius* (Anura, Hyperoliidae) in central République Démocratique du Congo. *Alytes* **24**, 40–60 (2006).

Schiøtz, A. Treefrogs of Africa. Addenda & Corrigenda 2007. Unpublished manuscript, available as pdf from http://zoologi.snm.ku.dk (accessed 27 July 2008), 1–14 (2007).

Shen, X. X., Liang, D., Feng, Y. J., Chen, M. Y. & Zhang, P. A versatile and highly efficient toolkit including 102 nuclear markers for vertebrate phylogenomics, tested by resolving the higher level relationships of the Caudata. *Mol. Biol. Evol.* **30:** 2235–2248 (2013).

Smith, S. A., Stephens, P. R. & Wiens, J. J. Replicate patterns of species richness, historical biogeography, and phylogeny in Holarctic treefrogs. *Evolution* **59**, 2433–2450 (2005).

Wiens, J. J., Fetzner, Jr. J. W., Parkinson, C. L. & Reeder, T. W. Hylid frog phylogeny and sampling strategies for speciose clades. *Syst. Biol.* **54**, 778–807 (2005).
